# LTC: a novel algorithm to improve the efficiency of contig assembly for physical mapping in complex genomes

**DOI:** 10.1186/1471-2105-11-584

**Published:** 2010-11-30

**Authors:** Zeev Frenkel, Etienne Paux, David Mester, Catherine Feuillet, Abraham Korol

**Affiliations:** 1 University of Haifa, Institute of Evolution, Haifa 31905, Israel; 2 INRA, Genetics, Diversity and Ecophysiology of Cereals, Clermont-Ferrand, France

## Abstract

**Background:**

Physical maps are the substrate of genome sequencing and map-based cloning and their construction relies on the accurate assembly of BAC clones into large contigs that are then anchored to genetic maps with molecular markers. High Information Content Fingerprinting has become the method of choice for large and repetitive genomes such as those of maize, barley, and wheat. However, the high level of repeated DNA present in these genomes requires the application of very stringent criteria to ensure a reliable assembly with the FingerPrinted Contig (FPC) software, which often results in short contig lengths (of 3-5 clones before merging) as well as an unreliable assembly in some difficult regions. Difficulties can originate from a non-linear topological structure of clone overlaps, low power of clone ordering algorithms, and the absence of tools to identify sources of gaps in Minimal Tiling Paths (MTPs).

**Results:**

To address these problems, we propose a novel approach that: (i) reduces the rate of false connections and Q-clones by using a new cutoff calculation method; (ii) obtains reliable clusters robust to the exclusion of single clone or clone overlap; (iii) explores the topological contig structure by considering contigs as networks of clones connected by significant overlaps; (iv) performs iterative clone clustering combined with ordering and order verification using re-sampling methods; and (v) uses global optimization methods for clone ordering and Band Map construction. The elements of this new analytical framework called Linear Topological Contig (LTC) were applied on datasets used previously for the construction of the physical map of wheat chromosome 3B with FPC. The performance of LTC vs. FPC was compared also on the simulated BAC libraries based on the known genome sequences for chromosome 1 of rice and chromosome 1 of maize.

**Conclusions:**

The results show that compared to other methods, LTC enables the construction of highly reliable and longer contigs (5-12 clones before merging), the detection of "weak" connections in contigs and their "repair", and the elongation of contigs obtained by other assembly methods.

## Background

Until very recently, genome sequencing projects such as the human ([1,2]), mouse ([3], rice ([4]), or maize genome projects ([5,6]) have relied on the construction of physical maps as a framework to support BAC-by-BAC or whole-genome shotgun sequencing [7]. Alternatively, genome sequencing could be conducted via whole genome shotgun approach ([8-11] etc.) or using the novel next generation sequencing technologies (e.g., [12-14]). Physical maps can be established via BAC clones fingerprinting using restriction enzyme profiling [15-22] or by digital fingerprinting [23-25], and subsequent assembly of the clones into contigs based on the systematic comparison of fingerprint profiles. BAC contigs are then ordered using molecular markers and genetic or radiation hybrid maps. In addition, to providing a framework for sequencing, such maps can be used for high-resolution gene mapping [26-28] and map-based gene cloning [29-34]. Despite significant progress in fingerprinting techniques such as High Information Content Fingerprinting (HICF) [22] and the development of efficient programs such as FPC (FingerPrinted Contigs) [35,36], maximal likelihood-based reconstruction of physical map [37,38], random cost algorithm minimizing triplewise linking distance [39], etc. to automatically cluster clones into contigs, physical mapping remains long, laborious, and expensive especially for large and complex genomes that contain a high amount of repeated sequences (e.g., maize or wheat genome). Therefore, the development of algorithms and methods making this process more cost effective is important in view of the increasing amount of non-model species that will be sequenced in the near future.

The basis of contig assembly is that the same DNA fragments in different clones are cut by a given restriction enzyme at the same sites. Hence, the presence of fragments with the same length in the fingerprints of two clones, *c*_1 _and *c*_2_, indicates a possible overlap between these clones. However, in large genomes, the abundance of repeated elements and the limited accuracy of scoring the band lengths may lead to the identification of shared bands for two clones that originate from different parts of a chromosome, thereby reducing the reliability of contig assembly. Thus, contig assembly relies on the identification of significant overlaps. This implies the calculation of p-values of clone overlap for any pair of clones *c*_1 _and *c*_2_. Namely, the p-value is the probability for two random clones to have the same or a higher number of shared bands by chance. The exact calculation of the p-value is usually problematic (reviewed in Wendl, 2005 [40]). The FPC software uses the Sulston approximation [17] that is based on the simplest model of taking into account tolerance (the accuracy of fragment length scoring), and the assumption that appearances of fragments (bands) of different lengths are independent and identically distributed (*iid *assumption). This approximation is valid in cases where a small number of clones are matching but can be very inaccurate in situations with an intermediate to large number of common bands [40,41]. Moreover, it was observed that different bands can have very different abundances within a fingerprint database. To take this variation into account Nelson *et al. *(2005) [6] proposed to exclude the most abundant bands for reducing the proportion of false overlaps. Other ways of taking into account band frequencies based on Bayes Theorem were also proposed (e.g., [42,43]), but such methods are cumbersome, especially with HICFs (e.g., [44]).

The FPC program package assembles clones into contigs based on fingerprints generated by either the end-labeled double digest method [15,18] or the complete digest method [16,45]. Because of technical difficulties related to the large amount of clones to order, FPC divides the clones into subsets of relatively small contigs in which clones are supposed to be highly significantly overlapping. Clones are ordered using local optimization and building band maps. To achieve a treatable size and ensure high accuracy of contigs, FPC users usually employ a very high initial threshold (cutoff) for the p-value of clone overlaps. However, for many clone pairs that do overlap physically, the p-value may not overcome such a cutoff. As a result, numerous short sub-contigs and singletons are produced calling for subsequent merging. In fact, the ordering of bands and highly overlapping clones in short contigs is questionable. The merging of short sub-contigs and singletons is also problematic.

Additional difficulties can be caused by the presence of "questionable" (chimerical or poorly fingerprinted) clones (referred to as Q-clones). The presence of Q-clones and false clone overlaps can result in a wrong clone order or even in the assembly of clones deriving from different parts of the genome into the same contig. The presence of bands with similar lengths also make a clone ordering complex thereby hampering the correct map assembly [46] and resulting in low-quality minimum tiling paths (MTP). In particular, unexpected gaps can arise when a MTP is checked by sequencing (e.g., via BAC end sequencing). Although many physical maps have been constructed with the standard FPC algorithm and successfully employed for genome sequencing, quite a lot of errors in contig assembling were also found [47]. The diversity of factors affecting the map quality in different situations calls for the development of new methods and tools complementing FPC and other existing packages. This problem is especially important for physical mapping of complex genomes with a high level of repeats, such as in wheat [48] and barley.

In this paper, we present a novel approach coordinating clone clustering and ordering. We also propose to use a new metric of clone overlap instead of the standard Sulston score. In contrast to FPC, the Linear Topology Contig (LTC, available upon request from the corresponding author) program starts clustering with a relatively relaxed cutoff and uses the topology of significant clone overlap to obtain longer contigs with a realistic (linear) structure. In each cluster, clones are ordered based on a global optimization procedure, and clones that disturb the order stability (assessed by re-sampling analysis) are excluded from the contig. Ordered contigs are then merged with a relaxed cutoff into longer contigs using the network representation of the significant clone overlaps as a control of the contig topology (similar ideas were used by Waterman *et al.*, 1986 [49]; Cuticchia *et al.*, 1992 [50]; Zhang *et al.*, 1994 [51] and others). In addition to contig building, LTC can be used for verification, repairing, and elongation of contigs obtained by other methods (e.g., FPC).

The reliability of the proposed methodology was assessed with HICF data from the wheat 3B physical map [48]. The performance of LTC vs. FPC was compared also on the simulated BAC libraries based on the known genome sequences for chromosome 1 of rice and chromosome 1 of maize. The results demonstrate that contigs built by LTC are longer, better ordered, and more robust to errors caused by false and missing bands than those obtained by FPC and can make the MTP selection more effective, leading to more reliable physical maps and increased sequencing cost efficiency.

## Methods

### I. Contigs Construction

In LTC, the contig construction algorithm includes the following steps: (i) calculation of p-values for clone overlaps; (ii) temporary exclusion from the analysis of the clones and clone overlaps unproved by parallel paths; (iii) adaptively changing cutoff clustering; (iv) "non-linear" cluster splitting into sub-clusters with linear topological structure; (v) global optimization ordering; (vi) verification of the orders by re-sampling; (vii) sub-contig merging into contigs; and (viii) MTP construction.

#### (i) Calculation of p-values for clone overlaps

In the first step, LTC calculates all pair-wise p-values *Pr*(*c*_1_, *c*_2_) of clone overlaps and selects threshold *Pr*_0 _(cutoff) to declare clones *c*_1 _and *c*_2 _with *Pr*(*c*_1_, *c*_2_) <*Pr*_0 _as overlapping clones. A proper choice of the threshold *Pr*_0 _should provide a reasonable trade-off between two requirements: (a) providing a sufficient number of pairs of overlapping clones, and (b) reducing the proportion of false overlaps among selected clone pairs. Instead of the Sulston score *Pr*^(*Sulst*) ^employed in the FPC package, LTC uses metrics *Pr*^(*Siid*) ^and *Pr*^(*Sind*) ^that estimate p-values more accurately (refer to Additional file 1, Section 1 for description of calculation) and the corresponding modifications *Pr*^(*SiidM*) ^and *Pr*^(*SindM*) ^that take into account the number of shared genetic markers. Metric *Pr*^(*Siid*) ^is based on the Sulston model of "random" clones [17]. This model assumes that appearances of fragments (bands) of different lengths are independent and identically distributed (*iid *assumption). Unlike the Sulston score, *Pr*^(*Siid*) ^provides a good approximation even in situations with relatively large clones and a high number of matching × bands. Metric *Pr*^(*Sind*) ^is based on a model with similar assumptions, but bands may not be identically distributed (in contrast to *iid *assumption) (for details see Additional file 1, Section 1).

Let *n*_1 _and *n*_2 _be the numbers of bands in clones *c*_1 _and *c*_2_. LTC approximates the probability that the number of bands present in both clones *c*_1 _and *c*_2 _is equal or higher than *k *by exp{-(*a*_0 _+ *a*_1_*k *+ *a*_2_*k*^2^)}, for *k *>*k*_0_. Coefficients *a*_0_, *a*_1_, and *a*_2 _are estimated by the Monte-Carlo method, i.e., by simulating a large number of pairs of random clones (with *n*_1 _and *n*_2 _bands) and scoring the "observed" number of shared bands (refer to Additional file 1, Section 1 for details); value *k*_0 _is defined by inequality: exp{-(*a*_0 _+ *a*_1_*k *+ *a*_2_*k*^2^)} < 0.001 for all *k *>*k*_0 _(see also Additional file 1, Section 2). Shared genetic markers are taken into account by adding the term *a*_mark_*m *to *a*_0 _+ *a*_1_*k *+ *a*_2_*k*^2^, where *m *is the number of shared markers. We used *a*_mark _= 100 ln 10 to make the overlap of clones with a shared marker more significant than 10^-100^. If bands are not assumed to be identically abundant, then the number of shared bands *k *is represented by -∑ ln*f*_*b*_, where the sum is taken over all shared bands and *f*_*b *_is the abundance of band *b*. Clearly, if all bands have equal abundances then *k *is proportional to the number of shared bands (i.e., both formulations will give the same result). Values *f*_*b *_can be estimated by the maximum likelihood method: the probability that a random clone with *n *bands contains band *b *is 1-(1-*f*_*b*_)^*n*^. [Logarithm of likelihood is then equal to ∑_*c *__with __*b *_log(1-(1-*f*_*b*_)^*n*(*c*)^) + ∑_*c *__without __*b *_log((1-*f*_*b*_)^*n*(*c*)^); for small *f*_*b *_(*f*_*b*_<<1/*n*_max_, *n*_max_= max_*c *_*n*(*c*)), the maximum likelihood estimation for *f*_*b *_is close to *n*_mean_^-1 ^*π*_*b*_/(1-*π*_*b*_), where *π*_*b *_is a proportion of clones in the entire database having band *b *and *n*_mean _as the mean number of bands in the clone]. Taking band abundances into account satisfies the additive condition, when the sum of weights of two bands is equal to the weight of "pair of bands" that appears with an abundance equal to the product of abundances of the bands (because bands are supposed to be independent).

#### (ii) Temporal exclusion of clones and clone overlaps not proved by parallel paths (TENPP-procedure)

In contrast to FPC, LTC excludes putatively false significant overlaps and putatively problematic clones before clustering. The main idea behind the identification of problematic clones and clone overlaps is that each part of the chromosome is most probably covered by several clones (although in fact, some parts can be uncovered or poorly covered by clones of the BAC library). One can expect that chimerical clones and false clone overlaps are not proven by parallel clones [39,52]. Thus, clustering should subdivide the clones into groups covering different parts of the chromosome. LTC clusters the clones in such a way that each position of a chromosomal region (without ends), covered by clones from the cluster, is covered by several (at least by three) significantly overlapping clones. Moreover, it requires that, even after excluding any single clone or clone overlap, for any pair of clones *c*_*i *_and *c*_*j *_from the cluster *C*_0_, a sequence of clones *c*_(1)_,..,*c*_(__*n*__) _from *C*_0 _should exist such that *c*_(1) _= *c*_*i*_, *c*_(__*n*__) _= *c*_*j *_and overlap of clones *c*_(__*k*__) _and *c*_(__*k*__+1) _is significant for all *k *= 1,..,*n*-1.

Let *Pr*_0 _be a liberal level of cutoff (we used 10^-12^≈10^-3^/*N*^2^, with *N*≈60,000 clones in our analyses). LTC calculates all pair-wise clone overlaps *Pr *and considers the results as a net of significant (relative to the selected cutoff *Pr*_0_) overlaps. For each edge *e *(significant overlap) of the net, parallel short paths (of 2-5 edges) connecting vertexes (clones) of edge *e*, but not going through edge *e*, are searched (see Additional file 2, Fig. AF2.1a). Edges having no such paths are temporarily excluded from the analysis. Then, the following procedure is applied to each vertex *c*_0_: for each pair of neighbor vertices *c*_1 _and *c*_2 _(connected with *c*_0 _by edge, i.e., *Pr*(*c*_0_,*c*_1_) <*Pr*_0 _and *Pr*(*c*_0_,*c*_2_) <*Pr*_0_), short "parallel" paths of 2-5 edges in this net, connecting vertices *c*_1 _and *c*_2_, but not going through vertex *c*_0_, are searched (Additional file 2, Fig. AF2.1b). Vertices having at least one pair of neighbors without such parallel paths are also temporarily excluded from the analysis. The excluded clones and overlaps can be used later to merge or elongate contigs (see below). For convenience, we refer to such a procedure of **T**emporal **E**xcluding from the analysis **N**ot proven by **P**arallel **P**aths as to the TENPP procedure with respective cutoff.

#### (iii) Clustering with "adaptively" varying cutoff

The ideal way to obtain a reliable contig assembly is to write down a full likelihood for the entire problem that allows a possibility for errors and repeats in data and find out all solutions with likelihood close to the maximum [37]. However, the expected length of band map for entire dataset is usually too long and finding maximal likelihood solution will be too hard even for modern computers. Moreover, estimating the probabilities included in the likelihood function is also not straightforward. Subdividing the data into clusters putatively covering non-overlapped parts of genome sequences by identification of putatively false clone overlaps and chimerical clones can simplify the problem [37]. In contrast to FPC that uses a stringent uniform cutoff LTC starts clustering with a liberal cutoff *Pr*_0 _by the single-linkage algorithm [53], and selects the resulting **r**easonably **s**ized clusters (rs-clusters). By "reasonably sized clusters", we arbitrarily (for certainty) define clusters with 6-500 clones. Actually, larger clusters also can be analyzed by the LTC program and subdivided into parts with linear topological structure (see below). Such a restriction was used to reduce the CPU time increasing cubically with number of clones in cluster. Additional reason for this restriction was the fact that the program Pajek [54] used for the visual control of the net structure of significant overlaps cannot represent large nets well. Using other programs for net visualization can help to overcome this problem. Artificial split of real contig can result in additional errors in ordering of obtained parts complicating their merging on the next steps.

**Figure 1:**
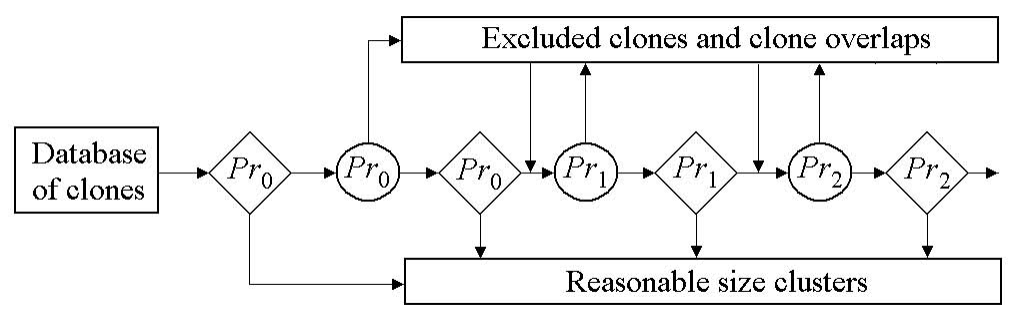
**Scheme of clone clustering with adapting cutoff**. Diamonds denote single-linkage clustering with corresponding cutoff. Circles denote procedure of excluding clones and clone overlaps not proved by parallel paths in the net of significant (relative to the corresponding cutoff) clone overlaps from the analysis.

For each cutoff level, LTC excludes putative false clone overlaps and putative chimerical clones in large clusters (with more than 500 clones) by the TENPP procedure described above. Then, the single linkage algorithm is run again. At the next step, LTC increases the stringency, but only after removing the selected reasonably sized clusters (i.e., protecting them from further "dissolving"). A schematic representation of the algorithm is provided in Figure 1. Refer to Additional file 1, Section 3 for simple example illustrating the efficiency of the proposed algorithm. Note that repeating the TENPP procedure will be effective only in cases of high proportions of false significant clone overlaps. If such proportion is not high, then it is reasonable to expect that most of the putatively chimerical clones and false clone overlaps will be excluded directly after the first TENPP procedure. Sizes of clusters can also be reduced by temporary exclusion of "buried" clones. These clones can be used later to obtain more reliable band maps.

#### (iv) Looking for linear topological structure

**Figure 2:**
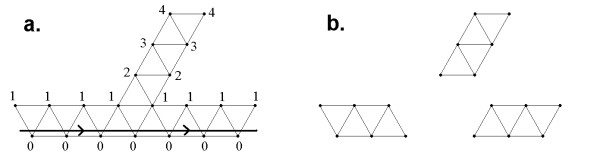
**Problematic clusters with non-linear topological structure**. **(a) **Identification of non-linearity of cluster topological structure: presence of vertices with not too small ranks (e.g., more than 2) points to non-short offshoots from the selected diametric path (marked in bold line). **(b) **Splitting clusters into sub-clusters having linear topological structure. See also Additional File 1, Section 4.

False clone overlaps and chimerical clones can lead to clusters with non-linear topological structures (Figure 2a) which is incompatible with the one-dimensional structure of eukaryotic chromosomes. To facilitate the detection and visualization of such clusters, LTC employs a representation of clusters as nets of significant overlaps. Obviously, ordering topologically non-linear clusters is problematic. To overcome this problem, we propose to split such clusters into sub-clusters with linear topological structure by excluding clones from the branching nodes from the analysis (Figure 2b). Non-linearity of the cluster structure can be detected by scoring ranks of vertices relative to the diametric path. By definition, a diametric path of the net is the longest, in terms of the number of edges, among the shortest paths between all possible pairs of vertices (e.g., [55]). Note that several diametric paths can exist for the same net. Any of diametric paths can be used for detection of non-linearity: The presence of vertices with rank 2 and higher indicates on non-short offshoots from the selected diametric path and, hence, a possibility of non-linear structure (Figure 2a).

#### (v) Ordering using global optimization

LTC orders clusters with linear topological structures without constructing their band maps. Here, the ordering problems are formulated in terms of global optimization of some criterion (similar to Fickett and Cinkosky, 1992 [56]; Alizadah *et al.*, 1993 [57]; Wang et al., 1994 [39]; Flibotte *et al.*, 2004 [46]). For simplicity, let's consider only the situation where all clones are not buried. This can be achieved by the temporal exclusion of buried clones, although sometimes this can lead to loosing the contig connection. The criterion *W*(Ω) of clone order Ω = (*c*_Ω(1)_,..., *c*_Ω(__*n*__)_) is calculated as:

$$W(\Omega )=\Sigma_{k}{}_{=\text{1},..,n-\text{1}}W_{\Omega \left(k\right),\Omega \left(k+\text{1}\right)}-b(\Omega )W_{0},$$

where *W*_*i*,*j *_= -log *Pr*(*c*_*i*_, *c*_*j*_), *b*(Ω) is the number of adjacent (within ordering Ω) clones with *Pr*(*c*_Ω(__*k*__)_, *c*_Ω(__*k*__+1)_) >*Pr*_0 _and *W*_0 _is the penalty for non-significant overlap of adjacent clones.

The maximization of such criterion can be reformulated as the well-known and intensively studied Traveling Salesman Problem (TSP) without the need to return to the starting point. Let *W*_max _be the maximum of *W*_*i*,*j *_= -log *Pr*(*c*_*i*_, *c*_*j*_). LTC defines distance between two clones as

$$d(c_{i},c_{j})=W_{\text{max}}-W_{i}{}_{,j}+W_{0}1\{Pr\left(c_{i},c_{j}\right)>Pr_{0}\},$$

where **1**{*Pr*(*c*_*i*_, *c*_*j*_) >*Pr*_0_} is indicator function equal to 1, if *Pr*(*c*_*i*_, *c*_*j*_) >*Pr*_0_, and equal to zero otherwise. Global optimization is effective especially when marker information is also available [46]. The exact solution to the TSP is a computationally challenging problem. Nevertheless, good heuristics (e.g., based on evolution strategy optimization) for the solution of TSP were developed for situations where the number of vertices is up to 10^3 ^orders of magnitude [58]. Coordinates of clone ends within the contig based on the solution path Ω_*best *_can be calculated by methods proposed by Flibotte et al. (2004) [46]. Using such global optimization approach can result in a reduced number of *Q*-clones and of places in the contig where two neighbor clones have an unexpected non-significant overlap. More effective triplewise linking distance also can be used to reduce the effect of false positive and false negative bands [39].

#### (vi) Resampling verification of ordering

The quality of the clone order within a contig is characterized not only by the value of the chosen criterion, but also by its robustness to small uncertainty of band content of the clones, which can be referred to as "contig stability". To evaluate this stability, LTC uses jackknife re-sampling iterations (in contrast to bootstrap ones used in Cuticchia et al., 1993 [59] and Wang et al., 1994 [39], that can artificially increase the significance of clone overlaps). Namely, LTC first constructs the order using clone overlaps scored over all bands. In addition, it constructs orders using clone overlaps based on randomly selected subsets of bands (say, 95% of the total set of bands). Then, the identification of unstable regions is conducted based on the frequency distribution of the right-side and left-side neighbors for each clone in the contig order. The higher the deviation from 1 (i.e., from the "diagonal" pattern) is, the less certain the local order is ([58,59]). One of the main reasons for the appearance of unstable orders is the high similarity of parallel clones that cannot be ordered properly due to missing and false bands. Excluding parallel clones allows the construction of a stable "skeleton" map, similarly to the approach suggested for building genetic maps (see [58]).

#### (vii) Merging of sub-contigs

After ordering, LTC tries to elongate contigs by merging those that display significant end-to-end overlaps (which may be also achievable by adding 1-2 supplementary intermediate clones), or by adding singletons. First, LTC re-analyzes all clones and clone overlaps temporally excluded at previous stages (see above). To elongate a concrete contig, LTC searches for all clones connected (by significant overlaps or via short paths of significant overlaps) with the clones from either of the contig ends. If adding all of the clones (for one of the two contig ends) does not lead to a violation of contig linearity, then such elongation is not problematic. If adding the clones does lead to branching (i.e., contradicts the linear structure of the chromosome), then each of the possibilities of linear elongations needs to be considered (e.g., see Additional File 2, Fig. AF2.2). The correct elongation can be detected by testing clone overlaps based on clone-end sequencing [23]. The same problem arises when clones from one contig significantly overlap with internal clones from another contig. The availability of DNA markers (in clones) with known chromosomal position helps to prevent the merging of contigs from different chromosomal zones. Contigs resulting from elongation should be reordered (see stage (v)).

#### (viii) MTP establishment

In the current version of LTC, MTP construction was based only on the topology of clone overlaps without building of the band map (in contrast to FPC). In our algorithms, we assume: (i) that MTP should include the terminal clones of the contig; (ii) the adjacent clones in MTP should significantly overlap (at the chosen threshold); and (iii) MTP should be of minimal length, i.e., include the minimal number of clones. To satisfy these conditions, we select clones for MTP from the diametric path of the net of the significant clone overlaps (see paragraph iv). In practice other criteria come into play, such as whether or not a particular BAC has a genetic marker on it and whether or not BAC end sequence is available. A further sophistication of the criterion may also the lengths of the clones (longer clones should be preferable) and overlaps with clones disturbing the contig linearity (presence of markers based on BAC-end-sequencing of such clones can clarify clone-overlaps).

### II. Verification and improvement of FPC contigs

LTC can be used also to verify and improve contigs obtained with other methods and tools. To test the quality of a contig and understand the underlying reason(s) for the assembly problem, the following procedure can be applied: (i) calculate all pair-wise probabilities of clone overlaps and represent the contig as a net of significant overlaps; (ii) test connections within this net; and (iii) test for topological linearity of the contig. If poor overlap (at the sequence level) of adjacent clones in the MTP occurs together with low significance of the clones' overlap, but the contig is connected and has a linear structure, then it makes sense to attempt fixing the contig by reordering the clones and selecting an alternative MTP. In this case, one needs to take into account that if some part of the analysis was already done for the old MTP, then it can be cheaper to select a MTP with common parts with the old one, rather than to repeat the analysis for the optimal MTP. If the detected problems in the MTP can be explained by non-connectivity or non-linearity of the contig structure, then the contigs must be split into connected parts each with linear structures. LTC first temporally excludes clones and clone overlaps not proven by parallel paths from the analysis (see above). After contig splitting, reordering and verification, LTC attempts merging the contigs by decreasing cutoff stringency or via returning back the previously excluded clones and clone overlaps. LTC also checks (whenever possible) that the added clones are not specified as belonging to other parts of the chromosome.

#### Comparison of clone partitions

Different clone partitions obtained with different clustering schemes were compared for the number of clones covered by reasonable size clusters and by direct comparisons of clusters. The comparison was performed by scoring the Rand index *R *[60] and its modification *R*' (see Additional File 1, Secttion 5). Additionally to these characteristics, the mean number *M *of clusters of the one clone partition overlaps with each of the other clone partition clusters was scored; if the two partitions are very similar, *M *should be close to 1. Two modifications of the *M *value, M* and M**, that reduce the role played by small clusters and singletons were also scored (see Additional File 1, Section 5).

#### Graphical representation of the cluster topological structure

Topological structures of clusters are represented by the net of significant (relative to some specified cutoff) clone overlaps. The nets were drawn using the publicly available program Pajek [54]. Draft pictures were obtained with the Kamada-Kawai algorithm for drawing undirected graphs [61] performed in the Pajek program.

#### Simulation of BAC libraries based on the known genome sequences

To test the effectiveness of contig assembly algorithms we simulated clone libraries and clone fingerprints based on known genome sequences (analogously to Xu et al., 2004 [62] and Krzywinski et al., 2007 [63], instead of using artificial genome sequences employed by other authors, e.g. Cuticchia et al., 1992 [50]; Soderlund et al., 1997 [35]). By *R*_enzyme _we denote the sequentially ordered set of *N *restriction sites *r*_*i *_corresponding to the chosen enzyme sequence (we used HindIII). For convenience, we supplement this set by *r*_0 _and *r*_*N*__+1 _corresponding to the start and end points of the sequence. Let *L *be the total length of the sequence. For each generating clone the program selects a start and end points from *R*_enzyme_. Index *i*_start _is selected randomly from 0 to *N *+ 1. Index *i*_end _calculated by *i*_start _+ *s h*, where *s *is equal to 1 or -1 with probabilities 0.5 (defines clone direction) and *h *is the integer part of a normally distributed random value with mean *a *and variance *σ*^2 ^(defines clone sequence length distribution). Values *a *and *σ*^2 ^are selected such that mean and standard deviation of clone length be about 120 kbp and 30 kbp, respectively (*a *= 120*λ*, *σ *= 30 *λ*, where *λ *= (*N *+ 1)/*L*). Chimerical clones were generated as union of regular clones, occurring with a probability *p*_chimer _(= 0.05 in our simulations).

Fingerprint for generated clones were defined by the set of bands of *K *types (corresponding to *K *used protruding-end restriction enzymes) presented in the clone; we used enzymes BamHI, EcoRI, XbaI and XhoI (*K *= 4) analogously to Ding *et al. *(1999) [21]. Presence of a band *b *with length *L*_*b *_and type *k*_*b *_is defined by the presence of sequence part of length *L*_*b *_bp bounded by the restriction site of enzyme *k*_*b *_from the one side and restriction site of one of the *K *enzymes mentioned above, or restriction site of blunt-end restriction enzyme (we used HaeIII) or clone end from the another side. In fact, sequence parts bounded by two different protruding-end restriction enzymes are twice included into fingerprint. Bands with length <*L*_min _= 50 bp or >*L*_min _= 500 were filtered out. Clones with number of bands *n *<*n*_min _= 50 or *n *>*n*_max _= 250 were also filtered out.

In the preparation of input data for the FPC and LTC programs we introduced noise into sequence length scoring to simulate errors in wet fingerprinting process. "Observed" band length was calculated by *L*_*b *_+ *err*, where *err *is a random value uniformly distributed within interval (-*ε*, *ε*) or within (-3*ε*, 3*ε*) with probabilities *p*_*ε *_= 0.90 and *p*_3__*ε *_= 1-*p*_*ε *_= 0.1 respectively. This imples that difference between two observations of the same band is less than constant tolerance value *t *with probability *τ *= *p*_*ε*_^2^(1-(1-*t*/2*ε*)^2^) + *p*_3__*ε*_^2^(1-(1-*t*/6*ε*)^2^) + 2*p*_*ε*_*p*_3__*ε*_(*t*/3*ε*). We used *ε *= *t/*2 = 0.2 implying *τ *= 0.94. To satisfy FPC format, band lengths were multiplied by 30 and rounded (hence tolerance *t *= 12). Type of band was taken into account by adding 0, 5000, 10000 or 15000 for the resulted band length for bands of type *k *= 1, 2, 3 and 4, respectively. Some bands of clones were excluded (with probability *p*_missing _= 0.05) to simulate false negatives caused by problems in PCR reactions. Automatic FPC assembly was conducted using cutoffs 10^-75 ^- 10^-45 ^with step × 10^5 ^and *DQer *for contigs having more than 10% Q-clones (with step × 10^9^) and *ReBuild *with corresponding cutoff if needed (similar to Paux *et al.*, 2008 [48]).

## Materials

To illustrate the advantages of the LTC analytical framework we employed the results obtained in the physical mapping of wheat chromosome 3B [48]. The corresponding database included High Information Content Fingerprinting results of 56,952 of BAC clones obtained from the chromosome 3B specific library. The initial input data for LTC included band lengths classified according to four dyes. For each dye, up to 4500 distinct points (band sizes) were obtained. Two bands were considered of the same if their sizes were within a tolerance = *t *(e.g., constant *t *= 4).

The elements of the LTC analytical framework were tested with fingerprinting data from two regions of chromosome 3B corresponding to clones located in the 3BL7-0.63-1.00 and 3BS1-0.33-0.55 deletion bins. Sixty-nine contigs comprising 3,606 clones that were found to belong to bin 3BL7-0.63-1.00 and 180 (8,167 clones) to bin 3BS1-0.33-0 [48] were used in the analysis. Using LTC, these 249 contigs were verified/corrected/extended; alternative contigs were also constructed and compared to the results obtained with FPC.

### Simulated BAC libraries

Simulated BAC libraries *Lib*_RiceChr1 _and *Lib*_MaizeChr1 _were constructed based on the known genome sequences *Seq*_RiceChr1 _and *Seq*_MaizeChr1 _for chromosome 1 of rice (45 Mbp, available at http://rgp.dna.affrc.go.jp/whoga/download.html.en, file chr01.fa.gz) and chromosome 1 of maize (300 Mbp, available at http://ftp.maizesequence.org/current/assembly, we used version that was downloaded at June 10, 2010), respectively. Basic characteristics of the libraries are summarized in table Table 1.

**Table 1:** Basic characteristics of simulated BAC-libraries

Characteristic	***Lib***_**RiceChr1**_	***Lib***_**MaizeChr1**_
Sequence length	45,064,769 bp	300,239,041 bp
*N*_BinEnds_/*L*_Bin_	12,570/3,585.1 bp	110,910/2,707.1 bp
*N*_BandEnds _(*L*_BandReal_)	179,050 (251.7 bp)	1,392,692 (215.6 bp)
*n*_Mean_/*n*_MeanObs_	113.0/112.7	136.8/129.0
*L*_Clone_/*L*_CloneObs_	133.3 kbp/138.1 kbp	132.0 kbp/134.5 kbp
*L*_BandObs_	1,225.9 bp	1,042.4. bp
*N*_Clones_/*N*_ClonesSimul_	4,417/5,000	29,924/35,000
*Coverage*	13.5	13.4
*N*_Ch _= *N*_ch2 _+ *N*_ch3 _+ *N*_ch4_	202 = 198 + 4 + 0	1,016 = 999 + 16 + 1

Here *N*_BinEnds _is the number of restriction sites corresponding to enzyme HindIII chosen for clone-end simulation; *L*_Bin _is the mean length of bin equal to distance between restriction sites corresponding to enzyme HindIII; *N*_BandEnds _is the number of restriction sites corresponding to the enzymes BamHI, EcoRI, XbaI, XhoI and HaeIII used for fingerprinting; *L*_BandReal _is the mean distance between restriction sites corresponding to these enzyme; *n*_Mean _is the mean number of bands (with length from 50 to 500 and colored end) per clone; *n*_MeanObs _is the mean observed number of bands (bands with different end-colors are taken twice, but among bands with the same color and length only one delegate is observed). *L*_CloneObs _and *L*_Clone _are mean lengths of clones (with and without chimerical clones respectively); *L*_BandObs _is the mean length of bands with length from 50 to 500 bp; *L*_BandMap _is the mean length of bands used for band map length calculation (equal to *L*_CloneObs_/*n*_MeanObs_); *N*_ClonesSimul _is the number of simulated clones; *N*_Clones _is the number of simulated clones with observed number of bands from 50 to 250; *N*_ch2_, *N*_ch3_, and *N*_ch4 _are resulted numbers of chimerical clones with number of bands from 50 to 250 and composed by union of 2, 3 and 4 parts, respectively.

## Results and Discussion

### Distribution of bands and clone lengths

In total, 30 to 275 bands were scored (out of 18,000 possible) for each of the *N *= 56,952 clones of the 3B BAC library (for more details of this data see [48]). The frequency of a band *b *was calculated as *π*_*b *_= *N*_*b*_*/N*, where *N*_*b *_is the number of clones containing bands with lengths different from *b *not more than the tolerance value. The bands showed quite variable abundances (Additional File 2, Fig. AF2.3a). In particular, one band was observed with *π*_*b *_= 0.93, one with *π*_*b *_= 0.78, six with *π*_*b *_from 0.2 up to 0.32, 220 with *π*_*b *_from 0.1 up to 0.2, 654 with *π*_*b *_from 0.05 up to 0.1, and others were observed in less than 5% of the clones. The minimal band abundance was 1.2%. We found more than 30 of the five-band combinations ("band haplotypes") present in multiple clones in a range of 1000-2271 (3.6% of total) clones, where *π*_*b *_of bands was in the range of 0.120 to 0.156, pointing to highly significant "linkage disequilibria" of the bands. This can result from physical overlap of considered clones and from repeats [6,64]. The distribution of clone length (scored as number of bands) was bimodal (Additional File 2, Fig. AF2.3b). The origin of this bimodality is in the construction of the BAC library itself. Three sub-libraries were constructed including large, medium, and small fragments (see [65]). Fingerprinting results revealed that the medium length sub-library was actually a mix of large and small inserts, and not medium inserts. This resulted in two distinct populations of clone length.

### LTC, a new algorithm for building contigs from fingerprinted clones

In this work we have developed a new analytical framework, LTC, for contig assembly of fingerprinted BAC clones that can be used as an alternative or as a complement to FPC. In contrast to FPC, the LTC program starts clustering with a relatively relaxed cutoff and uses the topology of significant clone overlaps to obtain longer and more realistic contig structures. Instead of a uniform cutoff, LTC uses a procedure that adaptively increases the cutoff stringency. Using stringent cutoffs only for large clusters generates fewer short contigs or singletons. After ordering, LTC merges the contigs by relaxing the cutoff (analogously to FPC), hence, this approach can be referred to as "up-down-up", in contrast to the FPC approach that can be referred to as "down-up" (stringent cutoffs applied from the beginning). LTC differs from FPC in a number of important features that are: (a) the metrics of clone overlap, (b) consideration of the band abundances, (c) the algorithm used for clone clustering, (d) the use of the topological structure of clusters for contig construction, (e) the application of global optimization methods for clone ordering, and (f) the assessment of the reliability of the mapping results by re-sampling. These parameters should enable the construction of more robust and longer contigs in particular when dealing with large and repetitive genomes.

To demonstrate the efficiency of LTC in contig assembly, we compared contigs obtained by LTC with contigs obtained using the standard FPC package for BAC clones from the wheat chromosome 3B [48]. The results are presented for the three main analytical stages shared between the LTC and FPC packages, i.e., (i) the identification of significant clone overlaps; (ii) the subdivision of clones into clusters of significantly overlapped clones; and (iii) the ordering of clones within clusters. To assess the capacity of LTC to verify, repair, and elongate contigs obtained by FPC, we also ran LTC on the 3B dataset previously obtained by FPC for the same contigs.

#### (i) Identification of significant clone overlaps by LTC

**Figure 3:**
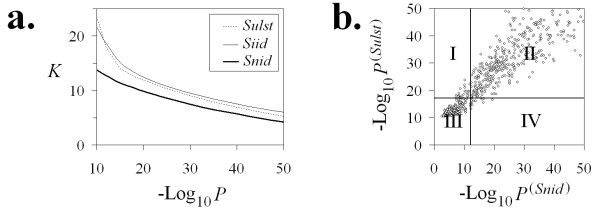
**Comparison of metrics**: **(a) **Number of significant clone overlaps *K *per clone for different cutoffs. Using liberal cutoffs *P*_0_^(*Snid*) ^= 10^-12^, *P*_0_^(*Sulst*) ^= 10^-17.2^, and *P*_0_^(*Siid*) ^= 10^-19.4 ^give about the same number of significant clone overlaps (~ 12.7 per clone). **(b) ***Pr*^(*Sulst*) ^vs. *Pr*^(*Snid*)^. Dots in rectangle I correspond to clone overlaps significantly relative to *Pr*^(*Sulst*) ^= 10^-17.2 ^but not significantly relative to *Pr*^(*Snid*) ^= 10^-12 ^(false positives). Dots in rectangle I correspond to clone overlaps significantly relative to *Pr*^(*Sulst*) ^= 10^-17.2 ^but not significantly relative to *Pr*^(*Snid*) ^= 10^-12 ^(false positives). Dots in rectangle IV correspond to clone overlaps significantly relative to *Pr*^(*Snid*) ^= 10^-12 ^but not significant relatively to *Pr*^(*Sulst*) ^= 10^-17.2 ^(false negatives).

Both FPC and LTC identify significant clone overlaps in a similar way: for each pair of clones, *c*_1 _and *c*_2_, the p-value *Pr*(*c*_1_, *c*_2_) of clone overlap is calculated and compared with a predefined threshold *Pr*_0 _(cutoff). Clones *c*_1 _and *c*_2 _with *Pr*(*c*_1_, *c*_2_) <*Pr*_0 _are declared as overlapping clones. In contrast to FPC that uses Sulston score *Pr*^(*Sulst*) ^[17], LTC uses new alternative metrics *Pr*^(*Siid*) ^and *Pr*^(*Sind*) ^(see paragraph (i) in Methods) for more accurate approximation of p-value. To compare metrics used in FPC and LTC, values *Pr*^(*Siid*)^, *Pr*^(*Sulst*)^, and *Pr*^(*Snid*) ^were calculated for each pair of clones from the 3B dataset (56,952 clones). The results indicate that *Pr*^(*Siid*) ^generally overestimates the significance of clone overlap compared to *Pr*^(*Snid*)^. For highly significant clone overlaps, *Pr*^(*Siid*) ^also provides a substantially higher *p*-value than the standard Sulston score *Pr*^(*Sulst*) ^(see Figure 3). However, logarithms of *Pr*^(*Siid*)^, *Pr*^(*Sulst*)^, and *Pr*^(*Snid*) ^were not absolutely correlated, especially for strongly overlapping clones. For example, liberal cutoffs *P*_0_^(*Snid*) ^= 10^-12^, *P*_0_^(*Sulst*) ^= 10^-17.2^, and *P*_0_^(*Siid*) ^= 10^-19.4 ^gave comparable numbers of significant clone overlaps (~ 12.7 per clone, see Figure 3a). We found that 2.1% of clone-overlaps, which was significant with *Pr*^(*Snid*)^, was not significant with *Pr*^(*Sulst*) ^(i.e., were identified as false positives) and vice versa (see Figure 3b). Similarly, 2.5% and 2.7% of false positives were found for pairs based on the *Pr*^(*Siid*) ^&*Pr*^(*Snid*)^, and *Pr*^(*Siid*) ^&*Pr*^(*Sulst*) ^criteria for clone overlaps, respectively. Such inconsistencies of the criteria can be explained by non-identical band abundances and by insufficient accuracy of the Sulston score to estimate clone overlap probability (see [40]).

A more accurate estimation of p-values for clone overlaps used in LTC reduces the proportion of falsely significant clone overlaps and increases the proportion of real clone overlaps that have a significant number of common bands. This leads to a reduction of clustering errors. Such errors can result in two undesired outcomes: (a) a wrong contig ordering with poor overlap of some adjacent clones from MTP, referred to as a *gap *that will call for (non-natural) splitting the contig into shorter contigs; and (b) a wrong partition of contigs into "independent" contigs that could not be merged. Both outcomes will yield shorter contigs than one could obtain using more correct clone clustering. Hence, using more accurate metrics for clone overlaps can result in longer contigs even with FPC algorithms for contig assembly.

Analogously to FPC, choosing cutoff stringency for clone clustering should be based on a tradeoff between the advantages of stringent and liberal cutoff values. Indeed, on the one hand, clustering with a liberal cutoff results in large but unreliable clusters, where ordering and detecting problematic clones and clone overlaps is challenging. On the other hand, clustering with a stringent cutoff results in many small clusters from which the ordering and identification of problematical clones is presumably "easy", while merging is difficult. Therefore, using liberal cutoffs may be a reasonable strategy if and only if powerful tools for multipoint ordering are available, such as with LTC.

#### (ii) Clustering the 3B fingerprinting dataset: comparing different procedures

##### Partition of clones using the LTC adaptive clustering procedure

**Figure 4:**
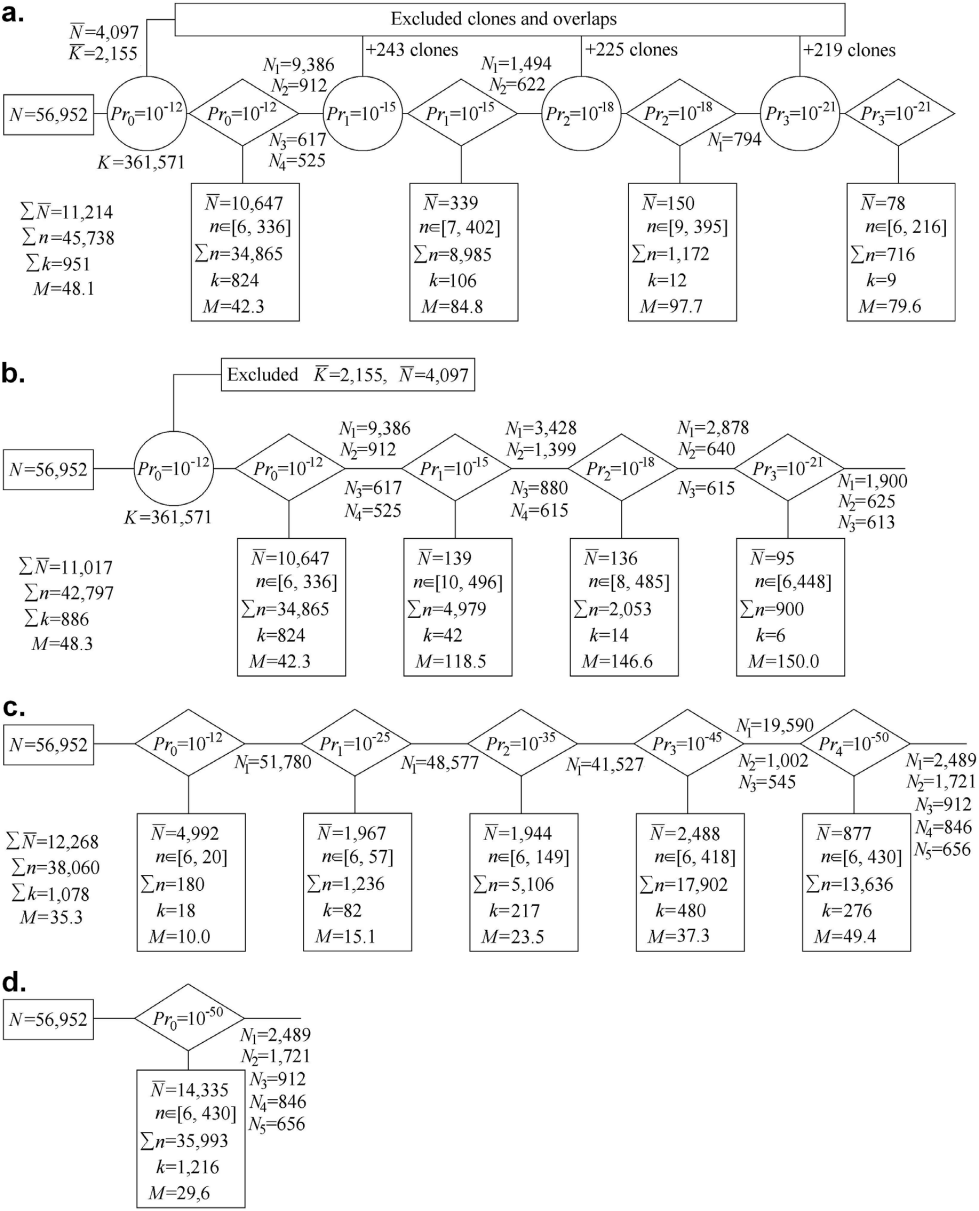
**Adaptive clustering using different scenarios**: **(a) **full scheme (see Fig. 1); **(b) **reduced scheme (TENPP procedure used only once); **(c) **clustering with adaptively changing cutoffs (for simplicity, only cutoffs 10^-12^, 10^-25^, 10^-35^, 10^-45^, and 10^-50 ^were used in the example); **(d) **clustering with uniform stringent cutoff 10^-50 ^(non-adaptive clustering analogous to FPC). Clustering (a) and (b) were based on *P*^(*SnidM*)^; clustering (c) and (d) were based on *P*^(*Snid*)^. *K *- number of significant clone overlaps; *n *- number of clones in clusters of reasonable size (from 6 to 500); ∑*n *- total number of clones in clusters of reasonable size; $\bar{N}$ - number of clones in clusters with less than 6 clones, singletons, or clones excluded by TENPP procedures; $\bar{K}$ - number of significant clone overlaps excluded by TENPP procedures; *k *- number of clusters of reasonable size; *M *= ∑*n*/*k *- mean number of clones in clusters of reasonable size; *N *= 56,952 is the total number of clones in the database; *N*_*i *_-number of clones in the large or intermediate size cluster *i*.

We applied the LTC main adaptive clustering procedure to the 56,952 fingerprints obtained for chromosome 3B [48]. Out of *N*(*N*-1)/2 = 1.6·10^9 ^possible clone overlaps, 361,571 (i.e., 0.02%) were significant at *Pr*^(*SnidM*) ^<*Pr*_0 _= 10^-12 ^(Figure 4a). From these, 2,155 clone overlaps (0.6% of the significant ones) and 4,097 clones (7.2% of total) have not been confirmed by parallel paths (Figure 4a). After temporal exclusion of these clones and clone overlaps, the entire database was subdivided into 828 clusters with a minimal number of clones per cluster of 6. In total, 6,550 clones (11.5% of total) were found in clusters with smaller sizes or as singletons. Among the 828 clusters, one was large (9,386 clones), three were of intermediate size (912, 617, and 525 clones) whereas the remaining ones included less than 336 clones per cluster (Figure 4a).

The TENPP procedure was used again on the entire database with cutoffs *Pr*_1 _= 10^-3^*Pr*_0 _= 10^-15 ^which found additional significant clone overlaps and 243 clones not proven by short parallel paths. After temporal exclusion of these clones and clone overlaps, four large and intermediate clusters were subdivided into 108 clusters with at least 6 clones per cluster. In this stage, additional 339 clones were found either in smaller size clusters, or as singletons, or were excluded by the last TENPP procedure. Among the remaining 108 clusters, one was large (1,494 clones), one was intermediate (622 clones), and the others contained up to 402 clones per cluster (Figure 4a).

Another round of the TENPP procedure with cutoffs *Pr*_2 _= 10^-3^*Pr*_1 _= 10^-18 ^identified additional significant clone overlaps and 225 clones not proven by short parallel paths. Large and intermediate clusters were subdivided into 13 clusters with a minimal number of clones per cluster of at least 6 after temporal exclusion. This resulted in 150 clones that were found in clusters with smaller sizes or as singletons, or that were excluded by the last application of TENPP procedure. Thirteen clusters were obtained with one intermediate containing 794 clones while the rest had up to 395 clones per cluster (Figure 4a).

A final TENPP procedure with cutoffs *Pr*_3 _= 10^-3^*Pr*_2 _= 10^-21 ^found additional significant clone overlaps and 219 clones not proven by short parallel paths. After temporal exclusion, the remaining clusters were subdivided into 9 clusters with 6 to 216 clones; 78 clones were found in clusters with smaller sizes and singletons or were excluded by the last TENPP procedure (Figure 4a).

Thus, in total, 56,952 fingerprints were subdivided into 951 clusters comprised of 6 to 402 clones; 11,214 clones were found in clusters with less than 6 clones, were singletons, or were excluded by the last TENPP procedures. The average number of clones per cluster was 48.3. The partition of clones obtained with this first approach will be referred hereafter as ***C***_LTC_.

##### Skipping some components of the LTC clustering algorithm

To demonstrate the power of the LTC adaptive clustering procedure, we skipped some of the clustering scheme components described above (Figure 4a). Three versions were considered: (i) using TENPP only once; (ii) clustering with adaptively changing cutoff stringency but without the TENPP procedure; and (iii) clustering with a uniformly stringent cutoff (analogous to the FPC algorithm).

(i) Figure 4b shows that if the TENPP is used only once (with cutoff *Pr*_0 _= 10^-12^) then in addition to reasonable size clusters, clustering with cutoff *Pr*_1 _= 10^-15 ^results also in two large (with 3,428 and 1,399 clones) and two intermediate size (with 880 and 615 clones) clusters. Repeated clustering with the more stringent cutoff *Pr*_2 _= 10^-18 ^gives additional reasonable size clusters plus one large cluster with 2,878 clones and two intermediate size clusters (with 640 and 615 clones) (Figure 4b). Further increase of the cutoff stringency up to *Pr*_3 _= 10^-21 ^splits these clusters into one large cluster with 1,900 clones and two intermediate size clusters (with 625 and 613 clones, Figure 4b). The clone partition obtained with this scenario was called ***C***_1_.

(ii) Without using the TENPP procedure (Figure 4c), one very large cluster is obtained even for very stringent cutoffs (e.g., 51,780 for *Pr*_0 _= 10^-12^, 48,577 for *Pr*_1 _= 10^-25^, 41,527 for *Pr*_2 _= 10^-35^, and 19,590 for *Pr*_3 _= 10^-45^). This clone partition was called ***C***_2_.

(iii) Clustering with a stringent uniform cutoff (10^-50^) (Figure 4d) resulted in more singletons and smaller clusters compared to clustering with adaptively changing cutoff (Figure 4c). The clone partition obtained with the uniform cutoff will be referred to as ***C***_3_, whereas the partition obtained with FPC (after ordering and merging) will be called ***C***_FPC_. In partition ***C***_FPC_, 41,295 of the 56,952 BAC clones were automatically subdivided into 1,995 contigs (with 6 and more clones) that were manually merged into 1,036 contigs with 6 to 290 clones per contig; the remaining 15,675 clones were found in clusters with less than 6 clones or were singletons [48].

Among the 951 clusters of ***C***_LTC _(Figure 4a) only 47 have non-linear topological structures, and 31 of them have only one branching point. The branching 47 non-linear clusters were split into about 171 "topologically linear" clusters. Note that among the clusters obtained with the other scenarios (Figure 4c-d) the proportion of clusters with non-linear topological structure was much higher (up to 70% in ***C***_3_, not shown). Thus, these results demonstrate that the TENPP procedure enables the use of much more liberal cutoffs to obtain clusters of reasonable size. Repeating the procedure with more stringent cutoffs helps to split large clusters into reasonable size clusters. Clustering with adaptively changing cutoffs helps to protect from "dissolving" the reasonably sized clusters obtained at liberal cutoff stringency under more stringent cutoffs (see Additional File 2, Fig. AF2.4).

##### Comparing LTC vs. FPC clustering

The ***C***_LTC _clustering contains more clones in the reasonable size clusters than ***C***_FPC_: 80% vs. 73% of the 56,952 clones. As described in the Methods section (paragraph iii), "reasonable size of clusters" were arbitrary (for certainty) defined as clusters with 6 to 500 clones; obviously, other ranges can also be successfully used. Although the number of clusters in ***C***_FPC _was less than in ***C***_LTC _after the split into clusters with linear topological structure (1,036 vs. about 1,200), ***C***_LTC _clusters are proven by parallel clones and hence should be more reliable. Moreover, many of the FPC clusters were obtained by manual merging smaller clusters, whereas 1200 clusters were obtained by LTC before any merging was undertaken. The Rand index was high, *R*(***C***_LTC_, ***C***_FPC_) = 0.997 because most of the clone pairs appeared in different clusters. The modification of *R*: *R*'(***C***_LTC_, ***C***_FPC_) = 0.56 was more informative, reflecting that large clusters in ***C***_LTC _and ***C***_FPC _were rather different (one of the FPC clusters overlapped with eight LTC clusters). Nevertheless, the values of *M*_1_(***C***_LTC_, ***C***_FPC_) = 3.7, *M*_1_*(***C***_LTC_, ***C***_FPC_) = 2.15, and *M*_1_**(***C***_LTC_, ***C***_FPC_) = 1.25 for the mean number of clusters from ***C***_LTC _overlapped with ***C***_FPC _(see Comparison of clone partitions in the Methods section) indicating that most of the differences between ***C***_FPC _and ***C***_LTC _originate from the difference between Q-clones excluded by FPC and those excluded by the TENPP procedure in LTC. If one excludes the clones and clone overlaps from the ***C***_FPC _clusters that were excluded by TENPP in LTC and a clustering is applied with a liberal cutoff *Pr*_0 _= 10^-12^, then a new clustering ***C***_FPC_^(TENPP) ^is obtained with *M*_1_**(***C***_LTC_, ***C***_FPC_^(TENPP)^) = 1.3 and *M*_1_**(***C***_FPC_^(TENPP)^, ***C***_LTC_) = 1.0. This demonstrates that each of ***C***_FPC_^(TENPP) ^clusters is actually a sub-cluster of ***C***_LTC_. Thus, all of LTC contigs are longer than the corresponding FPC sub-contigs proven by parallel clones.

#### (iii) Verification of FPC contigs

The LTC adaptive clustering procedure was also used to test 249 FPC contigs (each containing 6 to 290 clones; the corresponding clone partition was mentioned in the previous section and denoted by ***C***_FPC_) that were assigned to the 3BL7-0.63-1.00 and 3BS1-0.33-0.55 deletion bins of wheat chromosome 3B [48]. The LTC analysis indicated that 201 of the contigs (80%) had regions where the overlap between two adjacent clones was not significant with the *Pr*_0_^(*Siid*) ^= 10^-12 ^criterion and therefore were considered as gaps by LTC. The maximum number of gaps per contig was 44 (found in a contig of 283 clones). The gaps detected in the FPC-based contigs were of five main origins: **(i) **Contig consisting of two (or more) non-connected parts; **(ii) **Buried clones causing conflicts between requests of linear ordering and contig connectivity; **(iii) **Weak power of ordering tools in FPC based on local optimization; **(iv) **Non-synchronous utilization of information on shared markers and common bands; **(v) **Topologically non-linear structure of the contig, mostly caused by the presence of clones and clone overlaps not proven by short parallel paths.

##### Contig consisting of two non-connected parts

Only one contig located in the 3BL7-0.63-1.00 bin corresponded to this category. The net of significant clone overlaps for this contig consisted of two non-connected parts (even when p-value of clone overlaps was calculated using standard and the very liberal cutoff *Pr*_0_^(*Sulst*) ^= 10^-10^). These parts can be connected by adding only one clone (see Additional File 2, Fig. AF2.5). Such a situation may result from the exclusion of one or two connecting clones (3B_043_I24 and 3B_073_B21) presumably considered as Q-clones by FPC at the stage of cluster ordering.

##### Buried clones causing conflicts between ordering and contig connectivity

**Figure 5:**
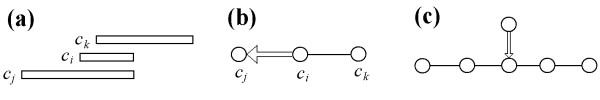
**Examples of situations where buried clones cause problems in clone ordering by LTC**. **(a) **Clone *c*_*i *_is buried in clone *c*_*j *_and significantly overlaps with clone *c*_*k*_, but overlap of clones *c*_*i *_and *c*_*k *_is not significant. **(b) **The net of significant clone overlaps for the situation (a). Exclusion of buried clone *c*_*i *_leads to a loss of connection between the left and right parts of the net. **(c) **Clones cannot be ordered to fit the requirement that adjacent clones overlap significantly (because the buried clone significantly overlapped with one clone only).

The significance of clone overlap is determined by the number of common bands and clone lengths. Hence, if clone *c*_*i *_is buried in clone *c*_*j *_and significantly overlaps with clone *c*_*k*_,, then it is not necessary that the overlap of clones *c*_*i *_and *c*_*k *_is also significant (see Figure 5a). Thus, after excluding buried clones, the net of significant clone overlaps can lose its connectivity (see Figure 5b). On the other hand, it is likely that each path along the net of significant connections, that visits all vertices, will visit the vertex corresponding to clone *c*_*i*_, buried in clone *c*_*j*_, before and after visiting the vertex corresponding to clone *c*_*j *_(see Figure 5c). In other words, vertex corresponding to clone *c*_*j *_is visited twice, and therefore, such paths cannot be found directly by tools constructed for solving the classical TSP. Such a situation was found in 12 contigs and could be solved by temporal exclusion of only a subset of buried clones from the cluster before ordering.

##### Weak power of ordering tools based on local optimization

Gaps that can be repaired by clone reordering within clusters were found in 198 of the 249 considered contigs (data not shown). Such reordering of clones usually changes clone positions in the contig only locally (see Additional File 2, Fig. AF2.6). Band maps based on LTC clone ordering were compared with FPC band maps. It was found that adjacent clones in LTC ordering cover on average more common bands in a corresponding band map than adjacent clones in the FPC band map (data not shown). This leads to better correspondence between number of common bands and number of common positions in the band map for significantly overlapped clones. Similar results were obtained by Flibotte et al. (2004) [46]. Increased coverage of the band map leads to higher robustness of LTC contigs to technical errors in fingerprinting. It also results in a lower number of band repeats. Hence, band maps based on LTC ordering are somewhat simpler than band maps obtained by FPC.

##### Topologically non-linear structure of the contig

**Figure 6:**
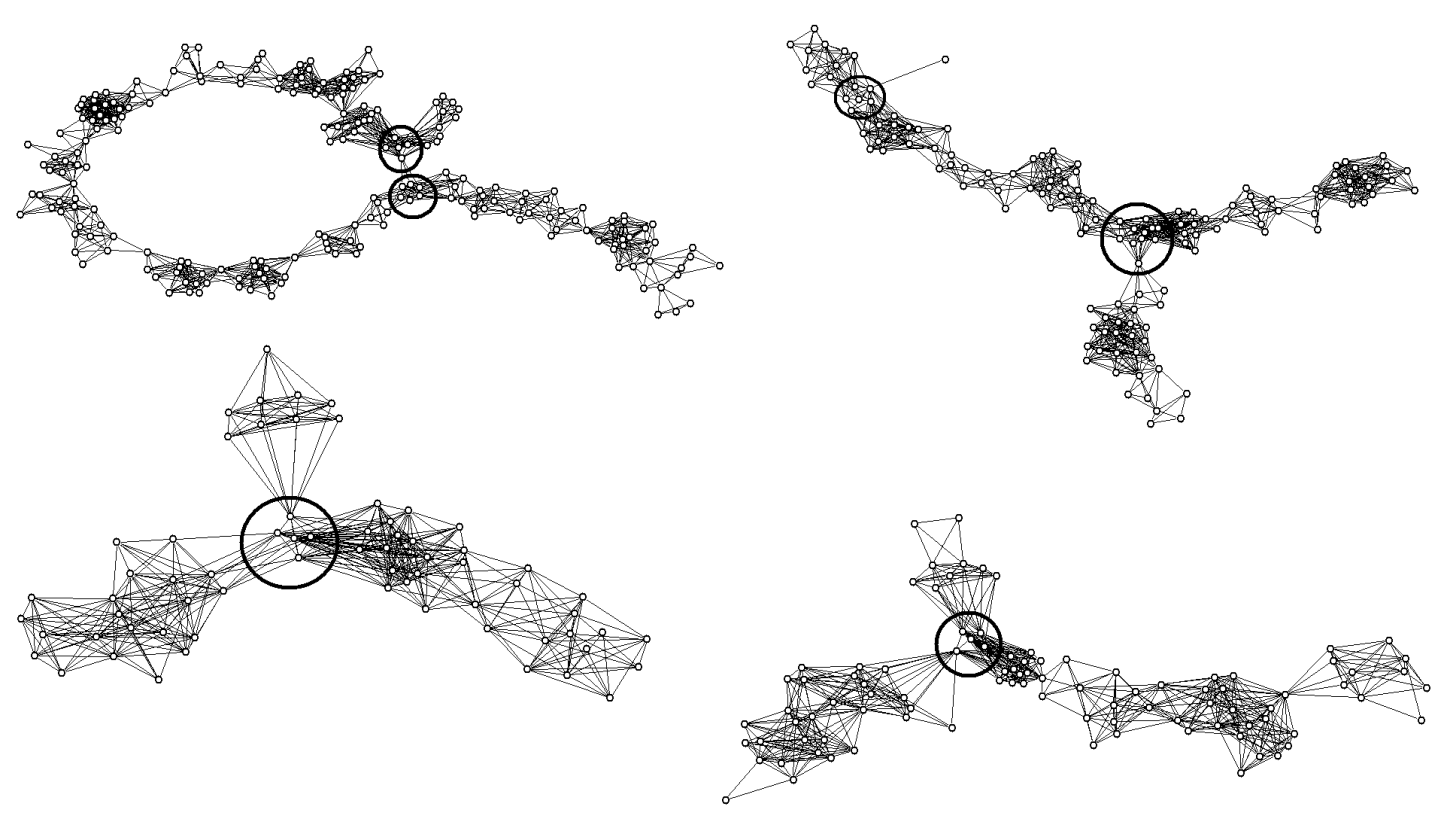
**Net representation of problematic FPC contigs with non-linear topological structure**. Presented four contigs from the 3BL7-0.63-1.00 and 3BS1-0.33-0.55 deletion bins display non-linear topological structures. Vertices represent clones; edges represent significant (relative to arbitrary selected cutoff *Pr*_0_^(*SnidM*) ^= 10^-12^) clone overlaps. Branching regions are marked by solid black circle.

A non-linear contig structure was found in 26 of the considered contigs (see examples in Figure 6). This was mostly due to the presence of clones and clone overlaps not proven by short parallel paths. Excluding such clones and clone overlaps (by TENPP procedure) and clustering by the single linkage algorithm subdivided all of the 26 contigs with non-linear structure into linear parts, but also splits some other (linear) contigs into smaller parts. Hence, some of LTC contigs are shorter than corresponding FPC contigs, but are much more robust to the presence of chimerical clones and false significant clone overlaps. It should be noted that end-to-end merging of LTC contigs seems to be a less problematic procedure than merging of FPC contigs because LTC does not place most problematic clones to the contig ends.

##### Non-synchronous utilization of information on common markers and common bands

In certain cases, the anchoring of markers to physical contigs leads to situations where a marker is found in two different contigs or even in two non-overlapping clones of the same contig. Such a situation can arise in cases when a single marker is situated on the overlap of two clones having only a non-significant number of common bands. It also can be a result of marker duplication or errors in marker amplification or contig assembly. Using FPC tools, such a situation cannot be recognized (because any pair of clones usually has several falsely common bands that can be erroneously considered as real overlap) and generally results in the artificial fusion of the two contigs. In contrast, LTC is able to detect such a situation because marker information is used synchronously with shared-band information in the analysis. In this case, the presence of duplicated markers and/or errors in marker amplification will usually lead to non-linearity in the topological structure of the cluster that will be detected by LTC (see Additional File 2, Fig. AF2.7).

#### (iv) Elongation of FPC contigs by using LTC

**Figure 7:**
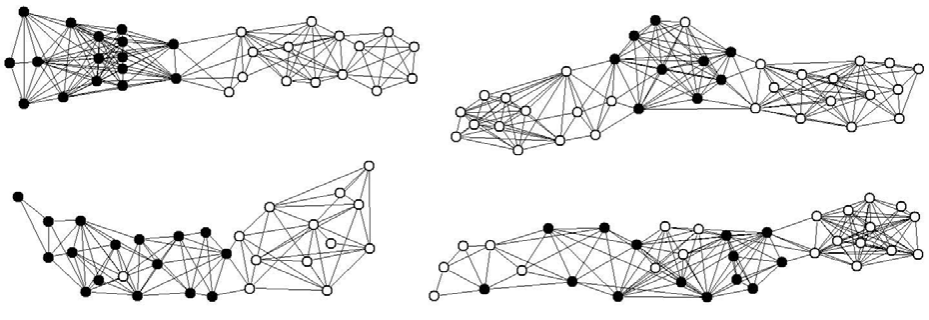
**Examples of de novo assembled contigs that proved to contain contigs constructed by FPC**. Black vertices reflect clones from the corresponding FPC contigs. White vertices correspond to clones which were not specified to belong to the zones 3BS1 or 3BL7.

FPC contigs from the 3BL7-0.63-1.00 and 3BS1-0.33-0.55 deletion bins were compared with the contigs that were constructed from the entire fingerprinting dataset using LTC (without initial clustering/ordering by FPC). It appeared that short FPC contigs (with 6-30 clones) were usually parts of LTC contigs. In such situations, LTC allowed the elongation of FPC contigs in a natural way. Several examples of such elongation of FPC contigs are presented in Figure 7. Long FPC contigs (with 50 and more clones) were usually different from those obtained with LTC. We assume that the main reason for such a difference is that clones and clone overlaps excluded by the TENPP procedure do not fully coincide with Q-clones and false significant clone overlaps identified by FPC. This leads to differences in clone partitions into clusters (see above) and can result in different contigs. In such cases, PCR amplification of BAC-end-sequences should be performed to assess the robustness of the contigs and determine which assembly is correct. For example, in the 3B dataset, some overlaps of adjacent clones from the MTP were not confirmed by sequencing and no satisfactory alternative MTPs could be found using FPC for these contigs (see Additional File 2, Fig. AF2.8a and b). For such situations alternative LTC contig assemblies were proven useful (e.g., to construct alternative elongation of the verified MTP (Additional File 2, Fig. AF2.8b). Note, sometimes LTC contigs included parts of different FPC contigs that were considered as belonging to different chromosomal zones based on marker assignment information (Additional File 2, Fig. AF2.9). We explain such observations by the fact that the location of the contig in the chromosomes is determined by position markers that were found in the clones of the contig. Hence, if contig assembly was made incorrectly, then the position of clones on the chromosomes is also determined with errors. Verification of the contig using BAC-end-sequencing can clarify the contig assembly and help to determine the position of clones on the chromosome.

### Comparison of LTC vs. FPC on simulated BAC libraries based on known genome sequences

Simulated BAC libraries *Lib*_RiceChr1 _and *Lib*_MaizeChr1 _were automatically assembled using FPC program and our LTC package (see sections Methods and Materials). Resulted contigs were tested using methods from LTC program and compared with real position of simulated clones in the genome sequence. Results of this analysis are briefly summarized in the Table 2.

**Table 2:** Contig assembly with FPC and LTC programs

	***Lib***_**RiceChr1**_		***Lib***_**MaizeChr1**_	
Statistic	FPC	LTC	FPC	LTC
*N*_ClonesCtg _*/N*_Clones _(%)	4,293/4,417 (97.2%)	4,231/4,417 (95.7%)	28,899/29,924 (96.6%)	28,994/29,924 (96.9%)
*N*_Ctgs_	42	25	257	190
*N*_MeanClonesCtg_	102.2	169.2	112.4	152.6
*L*_MeanClonesCtg_	1,045 kbp	1,731 kbp	1,128 kbp	1,532 kbp
*N*_ChCtg_/*N*_Ch_	183/202 (91%)	22/202 (11%)	946/1,016 (93%)	196/1,016 (19%)
*N*_QNotCh_/*N*_Q_	105/124 (85%)	106/186 (57%)	955/1,025 (93%)	110/930 (19%)
*N*_CtgsNonLinear_/*N*_Ctgs_	8/42 (19%)	-	23/257 (9%)	-
*N*_CtgCh_/*N*_Ctgs_	22/42 (52%)	0/25	108/257(42%)	0/190
*n*_CtgParts_	1.79	1.00	1.67	1.00
*N*_CtgOrdWrong_/(*N*_Ctgs_-*N*_CtgCh_)	4/20 (20%)	1/25 (4%)	47/149 (32%)	4/190 (2%)

Here *N*_ClonesCtg _is the total number of clones in contigs with 6 clones and more (others were excluded from the analysis); *N*_Ctgs _is the number of contigs with 6 clones and more; *N*_MeanClonesCtg _is the mean number of clones in the contigs with 6 clones and more; *L*_MeanClonesCtg _= *L*_CloneObs _*N*_MeanClonesCtg_/*Covarege *is estimated mean length of the contigs with 6 clones and more; *N*_ChCtg _is the number of chimerical clones in the contigs with 6 clones and more (false negatives in the procedure of Q-clone detection); *N*_QNotCh _is the number of non-chimerical clones that were identified as Q-clones (false positives); *N*_Q _is the number of clones that were not included into contigs; *N*_CtgsNonLinear _is the number of contigs with non-linear topological structure; *N*_CtgCh _is the number of contigs containing clones (except chimerical ones) from non-overlapping parts; *n*_CtgParts _is the mean number of non-overlapping parts per contig; *N*_CtgOrdWrong _is the number of continuous contigs with errors in clone ordering.

The results presented in Table 2 indicate that LTC contigs are longer and more reliable than those obtained by FPC: (a) average contig length 1,731 (LTC) vs. 1,045 kbp (FPC) for rice, and 1,532 vs. 1,128 kbp for maize; (b) number of contigs with non-linear topological structure was 0 out of 25 (LTC) vs. 8 out of 42 (FPC) observed in rice analysis, and 0 out of 190 (LTC) vs. 23 out of 257 (FPC) in maize. Low efficiency of chimerical clones identification seems to be the main source of the errors in the FPC contig assembly: only 9% (rice) or 7% (maize) of chimerical clones were not included into FPC contigs. Excluding clones and clone overlaps not proven by parallel clones (see also [52]) used in LTC algorithm enabled us to identify and exclude 89% (rice) and 81% (maize) of chimerical clones even using much more liberal cutoff: 10^-25^- 10^-30 ^(LTC) vs. 10^-45^-10^-75 ^(FPC). Diagnosis of contig linearity implemented in LTC can also assist in detecting problematic contigs assembled using FPC: 8 out of 22 chimerical rice FPC contigs and 23 out of 108 chimerical maize contigs were detected as having non-linear structure. LTC was also able to identify problems in clone positioning within FPC contigs leading to non-significant overlaps of adjacent clones. Reordering of clones within contigs without chimerical clones was usually able to fix these problems.

Using these simulated BAC-libraries we found that LTC can encounter some difficulties with ordering contigs that contain long repeats causing false significant overlap of clones that belong local neighborhoods. Likewise, we found one pair regions separated by ~27 Mbp in the sequence of maize chromosome 1 (positioned at 52 Mbp and 79.5 Mbp from the sequence start) with several significant (up to 10^-32^) overlaps between corresponding clones. In fact, these overlaps caused non-linear cluster topology (after TENPP procedure), but overlaps of clones within each of these two regions were by far more significant (10^-70^-10^-140^) and increasing the cutoff stringency resulted in split of this cluster into two with linear topological structure. LTC can also encounter difficulties in situations where chimerical clone consists of two parts that in fact belong to non-overlapping regions of a small neighborhood in the sequence. In addition, we found that most of chimerical clones that have not been detected by LTC actually consisted of one long part (100-150 kbp) belonging to the region of the contig and shorter part(s) (30-60 kbp) outside the region. We think that these chimerical clones can be identified by constructing more accurate maximal likelihood band map for LTC contig [37,38].

### General discussion

Although many physical maps constructed with the standard FPC algorithm have been successfully employed for genome sequencing, a substantial amount of errors were found in contig assembling [47] and subsequently corrected using complementary methods. Various factors, such as the genome composition (e.g., abundance of repeats), employed wet strategies and technologies of DNA cloning and clone fingerprinting, insufficient genome coverage of the BAC libraries, and a low accuracy of band scoring can affect the efficiency and accuracy of BAC assembly, hence the quality of the physical map. The power and flexibility of bioinformatics tools and human factors may also play a role: (i) from the beginning, reading the band sizes is somewhat subjective because it is very sensitive to selection of the threshold parameters for automatic peak detection; (ii) clustering (contig assembly) can be highly affected by false significant clone overlaps and chimerical clones; (iii) cluster ordering depends on the selection of an initial clone and the presence of equal-sizes but not common bands in different clones; (iv) decision-making in contig merging is difficult to formalize and the result depends on the choices made among a high number of possibilities; (v) anchoring (linking genetic and physical maps) is also mostly made by hand and verification of mapping results (at each stage) is needed.

Our results show that many of the previously mentioned difficulties can become less problematic with the use of new physical mapping algorithms and empowering of standard algorithms by additional tools. The quality of physical maps can be improved by a more accurate identification of chimerical clones and false clone overlaps. In particular this can be achieved by a more reliable scoring of the clone overlap p-value, by the utilization of "information content" of the bands, and by investigating the topology of nets of significant clone overlaps. Clustering can be improved by the utilization of clone overlaps with more liberal p-values: such overlaps result in larger clusters and hence simplify contig elongation and merging. Using more effective tools of contig ordering and ordering verification also improves contig quality and leads to shorter MTPs. More reliable multilocus genetic maps allow aligning the physical contigs to the correct chromosomal position more accurately thereby improving the quality of anchoring.

## Conclusions

The proposed LTC methodology helps in obtaining more realistic (relevant) clusters of clones that are expected to have linear topological structure corroborating the linear structure of the eukaryotic chromosome. Analytical tools for clone ordering based on global optimization methods allow achieving coordinated clone orders and, presumably, shorter band map. Tools of LTC can also be used to "cure" and elongate contigs obtained with FPC or other methods. In particular, the proposed framework proved effective for fixing gaps in MTPs detected at the sequence level as well as for choosing alternative MTPs to increase the efficiency of sequencing.

We have shown that the LTC program has a few advantages over standard FPC in contig assembly. The disadvantages of LTC are in its less friendly interface, which lead to the possibility to work only with a Windows operating system and to a rather primitive output of the results. Some steps, such as, the building of band maps and automatic merging of contigs, the possibility of compound alignment and optimization of physical and genetic maps, are still not implemented in LTC. At this stage, it would make sense to utilize the advantages of these two packages in the following way: (i) construct the contigs with LTC at the beginning of a physical mapping project; (ii) for each resulting contig separately construct band maps using FPC; (iii) test FPC orders using LTC; (iv) resolve the detected problems by excluding clones disturbing the LTC-order in FPC-contig; (v) select a MTP for the verified contigs; (vi) if some gaps are found in the resulting MTP, try to cure them by changing or adding clones to the MTP using LTC; (vii) align contigs to a consensus genetic map [66,67] using genetic markers using FPC. In addition, LTC can be used to check the results of physical maps already constructed by FPC and provide additional information or corrections by curing, elongating, and merging the FPC contigs. This can be useful in the final steps of a FPC-based project in which LTC can be used for selecting or curing the MTP.

Although our approximation of p-value seems to be more accurate than the standard Sulston score, better approximations are needed to estimate p-values for highly significant overlaps. This can be important for very large databases of clones (e.g., when mapping is based on genome-wide rather than chromosome-wide fingerprinting) especially for organisms with highly repeated genomes. To achieve stability of clone ordering we need to construct effective algorithms of excluding extra parallel clones for highly covered regions and buried clones without endangering contig connectivity. Ordering of clones is associated with the bands' relative positions in the chromosome. Hence, LTC procedure used for clone ordering can be improved by the parallel construction of a globally optimal map of bands. The process of merging contigs can be further optimized by better coordinating the "wet" and "dry" tools. We also think that criteria for the MTP selection can be improved by taking into account the number of bands and band abundances in clones and clone overlaps.

## Authors' contributions

ZF designed algorithms, wrote the code for LTC package, conducted the analysis using LTC (for all presented datasets) and FPC packages (for simulated data only) and wrote the manuscript. EP provided the HICF data on 3B, conducted the FPC analysis for 3B, suggested the algorithm for simulations, proposed ideas for comparison of LTC and FPC methodologies and participated in preparing the manuscript. DM developed algorithms and wrote code for fast global optimization of clones ordering within the contigs. CF contributed to the discussion and participated in preparing the manuscript. AK supervised the project, proposed ideas for LTC methodologies, contributed to developing of the algorithms, helped to conduct the analysis and wrote the manuscript. All authors read and approved the final manuscript.

## Supplementary Material

Additional file 1 **More detailed description of LTC approach**. We have included the following items in an additional file named add_file_1.doc: (i) Description of p-value approximation for clone overlapping; (ii) Paragraph about comparison of LTC-approximation of p-value for clone overlapping, Sulston score, and mutual overlap statistic; (iii) Example illustrating clustering with "adaptively" varying cutoff; (iv) Examples of complications that one can meet in dealing with clusters having non-linear topological structure; and (v) A paragraph about comparison of clustering results obtained by different methods. Click here for file

Additional file 2 **Supplemental figs**. In this file we present figures that were not included into the main text of the paper. These figures clarify ideas of LTC approach and illustrate some of our results. Click here for file
