# Supplementary material for: LTC: a novel algorithm to improve the efficiency of contig assembly for physical mapping in complex genomes
Source: BMC Bioinformatics. 2010 Nov 30;11:584. doi: 10.1186/1471-2105-11-584 (PMC3098104; doi:10.1186/1471-2105-11-584)
Supplement: Additional file 1 — More detailed description of LTC approach. We have included the following items in an additional file named add_file_1.doc: (i) Description of p-value approximation for clone overlapping; (ii) Paragraph about comparison of LTC-approximation of p-value for clone overlapping, Sulston score, and mutual overlap statistic; (iii) Example illustrating clustering with "adaptively" varying cutoff; (iv) Examples of complications that one can meet in dealing with clusters having non-linear topological structure; and (v) A paragraph about comparison of clustering results obtained by different methods. [file 1471-2105-11-584-S1.DOC]

**Supplemental materials**

**1. Approximation of p-value for clone overlapping**

During fingerprinting, every clone *c* can be naturally presented by the set of its distinguishable fragment lengths *B_c_*. The probability *Pr*(*c*_1_, *c*_2_) can be presented as a function *P*(*k*, *n*_1_, *n*_2_), where *k* is the amount of fragments with the same length, like clones *c*_1_ and *c*_2_, *n*_1_ and *n*_2_ are characteristics of clones *c*_1_ and *c*_2_. In the standard models *k*=*|B_c_*_1_∩*B_c_*_2_| is the number of common bands (matches), and *n*_1_=|*B_c_*_1_| and *n*_2_=|*B_c_*_2_| are the numbers of bands (distinguishable fragment lengths) in clones *c*_1_ and *c*_2_. Parameters of function *P*(*k*, *n*_1_, *n*_2_) are determined by the total number of possible fragment lengths, *L,* and methods that take tolerance into account (Wendl, 2005). It also depends on the model of random clone and additional assumption pointing to simplified calculations. For fixed *n*_1_ and *n*_2_, function *P*(*k*, *n*_1_, *n*_2_) exponentially decreases with increasing *k* for *k*>*k*_0_=*E*(*k*|*n*_1_, *n*_2_) (reviewed in Meyers *et al*., 2004). Our idea of the calculation of function *P*(*k*, *n*_1_, *n*_2_) is based on its approximation by function *F*(*k*)=exp{-(*a*_0_+*a*_1_ *k*+*a*_2_ *k*^2^)}, where values *a_i_*= *a_i_*(*n*_1_, *n*_2_) are estimated in numerical Monte-Carlo simulations corresponding to the selected model of random clones. To do that, we propose to develop an algorithm containing the following steps: **(i)** simulating *N*=10*^β^* pairs of clones (we used *β*=6-8); **(ii)** obtaining numerical estimation of *P*(*k*, *n*_1_, *n*_2_) for first several *k*: *f_k_*=*P*(*k*, *n*_1_, *n*_2_), *k=*0, 1,…,*k*_0_,…,*k*_max_(*β*) and **(iii)** calculating coefficients *a*_0_, *a*_1_, and *a*_2_ using linear regression -ln *f_k_*=*a*_0_+*a*_1_*k*+*a*_2_*k*^2^+*e_k_*, *k*=*k*_1_,…,*k*_2_, where *k*_1_=min(*k*: *k*>*k*_0_, *f_k_*<10^-^*^α^*), and *k*_2_=max(*k*: *f_k_*>10^-(^*^β^*^-1)^) (we used *α*>1 such that *k*_2_-*k*_1_≥4). Note that we do not expect that function *F*(*k*) will be a good approximation for *P*(*k*, *n*_1_, *n*_2_) in case of *k*<*k*_1_.

To make the calculation of the p-value for clone overlaps faster, we prepare, from the beginning, a table of coefficients *a*_0_, *a*_1_, and *a*_2_ for all possible pairs of clone lengths. It is also possible to use a table only for several characteristic clone lengths and estimate a minus logarithm of p-value using linear approximation. In particular, if the total number of bands is 18000 and the clones consist of 50-300 independent identically distributed bands, then using a table for clone lengths of 50, 100, 150, 200, 250, and 300, we can estimate the p-value up to one-order of magnitude accuracy relative to the table containing all possible clone lengths for p-values 10^-3^-10^-30^ (not shown). Such an estimation of p-value for clones *c*_1_ and *c*_2_ is designated as *Pr*(*^Siid^*)(*c*_1_,*c*_2_).

A similar way of p-value calculation can be used in a situation with relaxing the assumption of band distribution. Let band presence in clones be independent but not identically distributed (alternatively to the standard Sulston model where bands are identically distributed). In this case, we propose to use *k*=-∑*_b_* ln *f_b_* to measure the amount of fragments with the same length, like clones *c*_1_ and *c*_2_ (instead of previously used *|B_c_*_1_∩*B_c_*_2_|. Here the sum is taken over by all common bands *b*. Value *f_b_* is the probability of the presence of band *b*. It can be estimated by 1-(1-*π_b_*)^1/^*^n^*^_mean^, where *π_b_* is a proportion of clones in the entire database having band *b* and *n*_mean is a mean number of bands in the clone. Moreover, it seems to be reasonable to use *n*=-∑*_b_* ln *f_b_* (sum is taken over all bands of clone *c*) as one of characteristics of clone *c* (additionally to standard one |*B_c_*|), but we don't use it in the present work for simplicity). Such definition of *k* gives higher weight to rare bands and lower weight for frequent ones. The composed weight of the pair of bands *b*_1_ and *b*_2_ is equal to the weight of the band with a probability to be obtained equal to the probability of obtaining the pair of bands *b*_1_ and *b*_2_, which is equal to *f_b_*_1_*f_b_*_2_, because bands are considered to be independent. Analogously to the case of iid bands, we determine *P*(*^Snid^*)(*k*, *n*_1_, *n*_2_). In our tests clone with length *n* was simulated by the way analogies to fitness proportionate selection (selecting *n* individuals from *L* with fitnesses equal to *f_b_*) used in genetic algorithms and in studies of evolution in finite population models.

Alternatively, band frequencies can be taken into account directly by the modification of the formula for the Sulston score: instead of matching number *k* it can be used as a value for *k'*=-∑*_b_* (ln *f_b_*)/(ln *f*_mean_). Here the sum is taken over all shared bands and *f*_mean_=1/*L* is mean abundance of the band. Another way of taking band frequencies into account is to construct an analog of Sulston score with sums taken over all possible band combinations. Such a formula can be effectively calculated in the case when bands can be subdivided into a few (say 3-5) classes such that all bands in one class have exactly the same frequency. Although such generalizations are seemly interesting, we will not consider them in the present work because metric is only a detail of our analytical framework which can be realized even based on the standard Sulston score.

**2. Comparison of LTC-approximation of p-value for clone overlapping, Sulston score, and mutual overlap statistic**

We found that *Pr*(*^Siid^*) approximates the exact p-value of clone overlapping *Pr*(*^iid^*) (Wendl, 2005) corresponding to Sulston model, better than the Sulston score *Pr*(*^Sulst^*) itself (see Fig. AF1.1). It also can be effectively calculated even in the case of huge numbers of different possible bands, where the calculation of the exact p-value is very (up to impossible) laborious (Wendl, 2005; 2007). We also found that coefficients *a*_0_, *a*_1_, and *a*_2_ are inversely proportional to (*n*_1_*n*_2_)^2^ (see Fig. AF1.2), hence *Pr*(*^Siid^*)=*F*(*k*, *n*_1_, *n*_2_) can be considered as a generalization of mutual overlap statistic *μ*=*k*^2^/*n*_1_*n*_2_. This statistic *μ* was compared with some threshold *μ*_0_ for decision about clone overlap (Olson *et al*., 1986). This method was under strong criticism (e.g., Wendl, 2005) because the *μ*_0_ value was supposed to be constant for all experimental designs. In contrast to this method, our calculation of *Pr*(*^Siid^*) provides threshold values specified for the experiment designs. We found also that *kμ*^0.5^=*k*^2^/(*n*_1_*n*_2_)^0.5^ is proportional to the logarithm of the Sulston score for large *k* (see Fig. AF1.3).


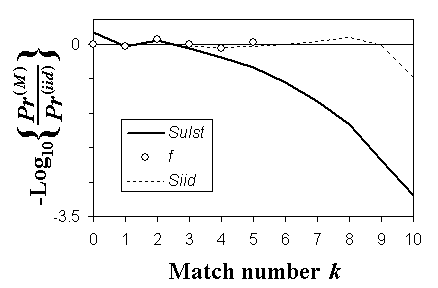


**Figure AF1.1. Comparison of the exact model of random clone overlap with its approximations.** Graph demonstrates that proposed *Pr*(*^Siid^*) approximates the exact *Pr*(*^iid^*) much better than the standard *Pr*(*^Sulst^*), which is very conservative. Parameters of the model: gel length *L*=235 bands, clone lengths *n*_1_=*n*_2_=10 bands. Values for *Pr*(*^iid^*) are taken from Wendl (2005). Values for *Pr*(*^Siid^*) were estimated by simulating *N*=10^8^ pairs of clones, *k*_0_=3, *k*_max_=5 (see Section 1).


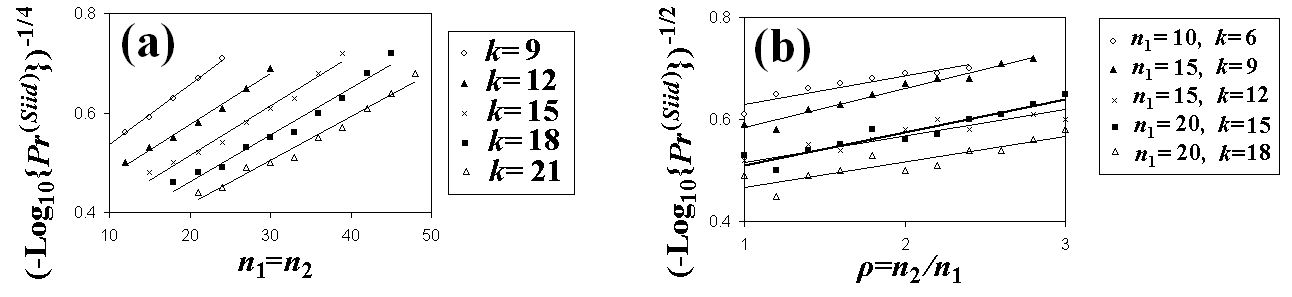


**Figure AF1.2. Dependence of p-value of clone overlapping (according to *Pr*(*^Siid^*)) on clone lengths.** **(a)** Clone lengths *n*_1_ and *n*_2_ are equal, match number *k*=*const*; **(b)** *n*_2_=*ρn*_1_, *ρ* rise from 1 to 3, *n*_2_=*const*, *k*=*const*. Observed linear dependence for various parameter values means that –log_10_*Pr*(*^Siid^*) is inversely proportional to (*n*_1_*n*_2_)^2^. Gel length *L*=235 bands.


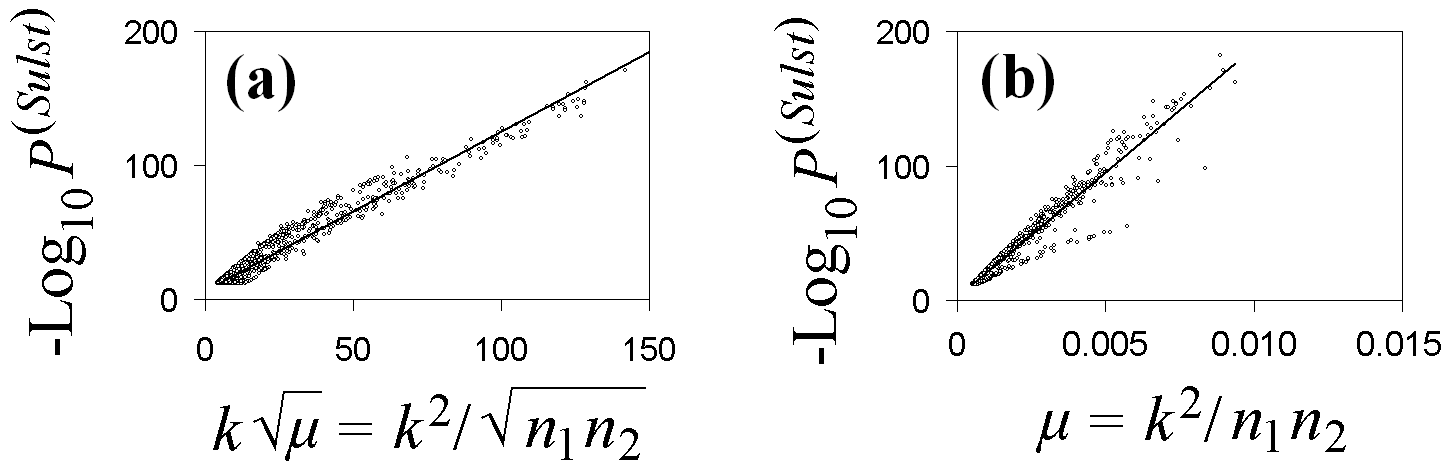


**Figure AF1.3. Sulston score *P*(*^Sulst^*) and mutual overlap statistic *μ*=*k*^2^/*n*_1_*n*_2_.** **(a)** Demonstration that -Log*P*(*^Sulst^*) is proportional to *kμ*^0.5^ = (*n*_1_*n*_2_)^0.5^*μ* = *k*^2^/(*n*_1_*n*_2_)^0.5^ for large *k*. **(b)** Demonstration that direct use of *μ* instead of the Sulston score is problematic for a linear approximation of the logarithm of Sulston score in the case of large *k*. In particular, higher *n*_1_*n*_2_ result in lower coefficient of proportionality. Gel length *L*=2,250 bands.

**3. Illustration of clustering with "adaptively" varying cutoff**

The efficiency of LTC clustering algorithm can be illustrated on a simple example of clone overlaps presented in Fig. AF1.4a. In this example, in order to simplify the explanations, we consider a situation with extremely small sizes of rs-clusters (2-4 clones only). For convenience, each clone from set *C* is named by its “coordinates” in Fig. AF1.4a. Single-linkage clustering algorithm with cutoff *Pr*_0_ subdivides the set of clones, *C*, into two clusters: *C*_0_^(I)^={(0,0), (0,1), (1,0), (1,1)} and *C*_0_^(II)^ that consists of all other clones from *C*. Cluster *C*_0_^(I)^ is already of reasonable size and thus can be protected. TENPP procedure with cutoff *Pr*_0_ excludes from the analysis clone overlap (3,3)(4,4) and clone (2,3). Single-linkage clustering of *C*_0_^(II)^ with cutoff *Pr*_0_ produces clusters *C*_0_^(III)^={(0,2), (0,3), (1,2), (1,3)}, *C*_0_^(IV)^={(4,2), (4,3), (5,2), (5,3)}, and *C*_0_^(V)^=*C*_0_^(II)^\(*C*_0_^(III)^*C*_0_^(IV)^(2, 3)). Clusters *C*_0_^(III)^ and *C*_0_^(IV)^ are already of reasonable size and can also be protected. TENPP procedure with cutoff *Pr*_1_ excludes clone overlap (3,2)(3,3) and clone (2,3) from the analysis. Note that clone (2,4) is still not excluded from the analysis because clone (2,3) is temporally returned for the next application of TENPP procedure. Further single-linkage clustering of *C*_0_^(V)^ with cutoff *Pr*_1_ produces clusters *C*_1_^(I)^={(4,0), (4,1), (5,0), (5,1)}, *C*_1_^(II)^={(2,1), (2,2), (3,1), (3,2)}, *C*_1_^(III)^={(4,4), (4,5), (5,4), (5,5)}, and *C*_1_^(IV)^={ (0,4), (0,5), (1,4), (1,5), (2,4), (2,5), (3,3), (3,4), (3,5)} and singleton (2,0). Clusters *C*_1_^(I)^, *C*_1_^(II)^, and *C*_1_^(III)^ are already of reasonable size and can be protected. Clone overlaps (2,3)(2,4) and (2,3)(3,3) are not significantly relative to cutoff *Pr*_2_, hence TENPP procedure with cutoff *Pr*_2_ excludes clone overlap (1,4)(2,3) and clone (2,4) from the analysis. Further single-linkage clustering of *C*_1_^(IV)^ with cutoff *Pr*_1_ produces clusters of reasonable size *C*_2_^(I)^={(0,4), (0,5), (1,4), (1,5)} and *C*_2_^(II)^={(2,5), (3,3), (3,4), (3,5)}. The resulting clusters are shown in Fig. AF1.4b.

Note that without application of TENPP procedure, the consequent use of single-linkage clustering with cutoffs *Pr*_0_, *Pr*_1_, and *Pr*_2_ to set of clones *C* gives quite a different result: single-linkage clustering of *C*_0_^(II)^ with cutoff *Pr*_1_ produces clusters *C*_1_^(V)^={(4,4), (4,5), (5,4), (5,5)}=*C*_1_^(III)^, set of singletons *S*={(0,2), (0,3), (2,0), (4,2), (5,2), (5,3)} and large cluster *C*_1_^(VI)^=*C*_0_^(II)^\(*C*_0_^(V)^*S*). Further single-linkage clustering of *C*_1_^(VII)^ with cutoff *Pr*_2_ produces clusters *C*_2_^(III)^={(2,1), (3,0), (4,0), (4,1)} and *C*_2_^(IV)^={(0,4), (0,5), (1,2) , (1,3) , (1,4), (2,3), (2,4), (2,5), (3,2), (3,3), (3,4), (3,5), (4,5)}, and singletons (2,2), (3,1), (5,0) and (5,1). The result of such a clustering procedure is shown in Fig. AF1.4c.

**
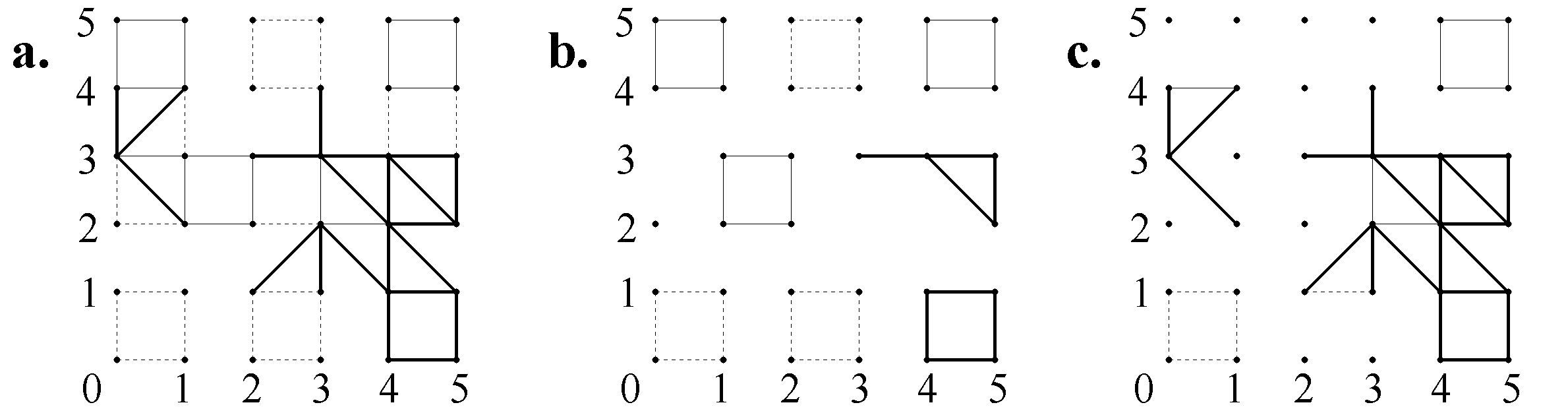
**

**Figure AF1.4. Net of clones and significant clone overlaps for considered example of clone clustering with adapting cutoff.** Vertices correspond to clones. Edges marked by dashed lines correspond to clone overlaps significant relative to cutoff *Pr*_0_, but not significant relative to the more stringent cutoff *Pr*_1_. Edges marked by solid lines correspond to clone overlaps significant relative to cutoff *Pr*_1_ (and hence relative to cutoff *Pr*_0_). **(a)** Initial net of significant clone overlaps. **(b)** Nets of significant clone overlaps in clusters obtained following the scheme presented in Fig. 1 in the main text of the paper. **(c)** Nets of significant clone overlaps in clusters obtained without use of TENPP procedure.

**4. Some complications in dealing with clusters having non-linear topological structure**

Maximal rank can depend on selection of diametric path (up to twice, see Fig. AF1.5a). Hence, the formulated criterion for non-linearity detection is sufficient but not necessary. Clones and overlaps causing non-linearity are excluded from the analysis only at the stage of sub-contig ordering although some of them may be used later for the merging of sub-contigs into a contig. It is possible that the net of significant clone overlaps for clones from a real contig will have non-linear topological structure due to false significant overlaps of non-adjacent clones (see example in Fig. AF1.5b). Moreover, even if the maximal rank of clones relative to some diametric path is one, it is possible that clones (even after excluding buried ones) could not be ordered in such a way that adjacent clones will significantly overlap. An example of such a situation is given in Fig. AF1.5c).


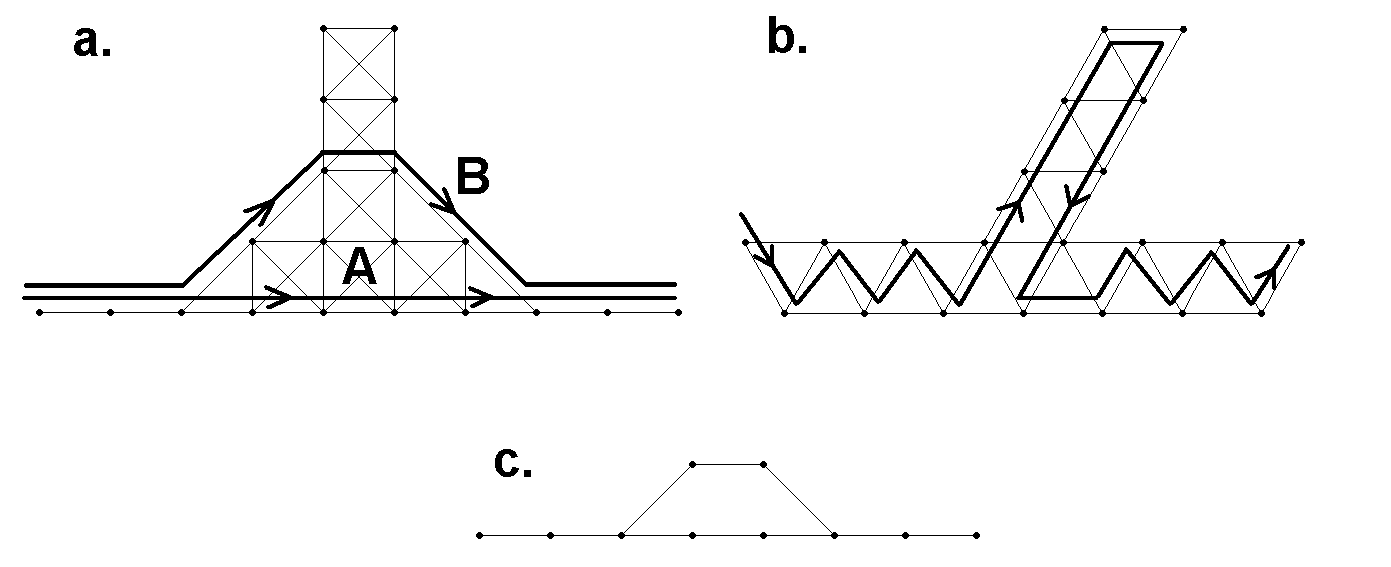
**Fig. Figure AF1.5. Examples of questionable clusters. (a)** Example of a net with maximal rank of vertex depended on the selection of a diametric path: for diametric paths A and B maximal ranks are four and two, respectively. **(b)** Example of a path visiting all vertices in the ambivalent structure. **(c)** Example of a net with maximal rank of vertices relative to a diametric path being no more than one, with no paths going through edges and visiting each of the vertices exactly once.

**5. Comparison of clustering results obtained by different methods**

Here, we present a possible way to compare different methods of clone clustering, e.g., the FPC clustering and our scheme described in sections Methods (*ii-iv*) in the main text of the paper. The difference between two data clusters ***C***^(1)^=(*C_i_*^(1)^)*_i_*_=1,.._,*_N_* and ***C***^(2)^=(*C_i_*^(2)^)*_i_*_=1,.._,*_N_*, where *N* is the number of clones in the database and *C_i_* is the number of clusters that contain element *i*, which can be scored by the Rand index (Rand, 1971). Let *t_ij_*(*^k^*)=**1**{*C_i_*(*^k^*)=*C_j_*(*^k^*)} be 1 if elements *i* and *j* belong in the same cluster in clustering ***C***(*^k^*) (*k*=1 or 2), and be 0 otherwise. Let *a*_11_=∑*_i_*,*_j_* *t_ij_*^(1)^*t_ij_*^(2)^, *a*_10_=∑*_i_*,*_j_* *t_ij_*^(1)^(1-*t_ij_*^(2)^), *a*_01_=∑*_i_*,*_j_* (1-*t_ij_*^(1)^)*t_ij_*^(2)^, and *a*_00_=∑*_i_*,*_j_* (1-*t_ij_*^(1)^)(1-*t_ij_*^(2)^). The Rand index is defined as *R*=(*a*_11_+*a*_00_)/(*a*_11_+*a*_10_+*a*_01_+*a*_00_) and has a value between 0 and 1. *R*=0 indicates that the two data clusters do not agree on any pair of points; *R*=1 indicates that the compared clusterings are exactly the same. Excluding elements from the analysis (e.g., clones unproved by parallel paths, Q-clones, singletons, and clones from clusters with very small number of clones) can be taken into accont by considering such elements as situated in trival clusters containing only one element.

In physical mapping the number of clusters is usually large, hence, for most of possible clone pairs, clones will be situated in different clusters, i.e., *t_ij_*(*^k^*)=0. Therefore, *a*_00_ is much higher than *a*_11_+*a*_10_+*a*_01_ and the Rand index *R* will be close to one even for very different data clusters. Alternatively, we can use a modification of the Rand index *R*'=*a*_11_/(*a*_11_+*a*_10_+*a*_01_). The shortcoming of such statistics is that it is very sensitive to cluster sizes, and large clusters give a much higher contribution than the small ones. To overcome these difficulties, we propose to complement the Rand index by the mean number of clusters from ***C***^(1)^ overlapping with clusters from ***C***^(2)^: *M*_1_(***C***^(1)^,***C***^(2)^)=1/|***C***^(1)^| ∑*τ*(*C*^(1)^, *C*^(2)^). Here |***C***(*^k^*)| denotes the total number of clusters in the data clusters ***C***^(1)^; the sum is taken over all cluster pairs (*C*^(1)^, *C*^(2)^); value *τ*(*C*^(1)^, *C*^(2)^) is equal to one if clusters *C*^(1)^ and *C*^(2)^ have at least one common element and equal to zero otherwise. The role of small clusters and singletons can be reduced (i) by uniting all of them into one "heap" cluster or, alternatively, (ii) by excluding them from the calculation. The corresponding value of *M*_1_(***C***^(1)^,***C***^(2)^) will be then designated as *M*_1_^*^(***C***^(1)^,***C***^(2)^) or *M*_1_^**^(***C***^(1)^,***C***^(2)^).

**References**

Meyers, B.C., Scalabrin, S., Morgante, M. 2004. Mapping and sequencing complex genomes: Let’s get physical! *Nat Rev Genet* **5:** 578–588.

Olson, M.V., Dutchik, J.E., Graham, M.Y., Brodeur, G.M., Helms, C., Frank, M., MacCollin, M., Scheinman, R., and Frank, T. 1986. Random-clone strategy for genomic restriction mapping in yeast. *Proc. Natl. Acad. Sci*. **83:** 7826–7830.

Rand, W.M. 1971. Objective criteria for the evaluation of clustering methods. *Journal of the American Statistical Association* **66**: 846–850.

Wendl, M.C. 2005. Probabilistic assessment of clone overlaps in DNA fingerprint mapping via a *priori* models. *Journal of Computational* *Biology* **12(3):** 283-297.

Wendl, M.C. 2007. Algebraic correction methods for computational assessment of clone overlaps in DNA fingerprint mapping. *BMC Bioinformatics* **8**:127.
