# Supplementary material for: LTC: a novel algorithm to improve the efficiency of contig assembly for physical mapping in complex genomes
Source: BMC Bioinformatics. 2010 Nov 30;11:584. doi: 10.1186/1471-2105-11-584 (PMC3098104; doi:10.1186/1471-2105-11-584)
Supplement: Additional file 2 — Supplemental figs. In this file we present figures that were not included into the main text of the paper. These figures clarify ideas of LTC approach and illustrate some of our results. [file 1471-2105-11-584-S2.DOC]

**Supplemental figures**


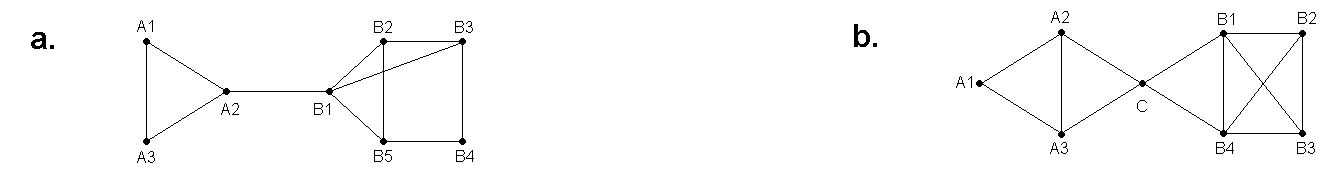


**Fig. AF2.1. Clones and clone overlaps not proved by parallel paths.** Set of significant (relative to the selected cutoff *Pr*_0_) clone overlaps considered as net: vertices correspond to clones, pair of vertices connected by edge if overlap of respective clones is significant. **(a)** Edge (clone overlap) A2B1 is not proved by parallel paths. All other edges are proven: for example, edge A1A2 proven by path A1A3A2, edge B1B3 proven by paths B1B2B3 and by B3B4B5B1. **(b)** Vertex (clone) C is not proved by parallel paths: all paths from A2 to B1 go through vertex C. All other vertices are proven: for example, vertex A3: path A1A3A2 proven by A1A2, path A2A3C proven by A2C, path A1A3C proven by A1A2C.

Note that an excluded edge is not necessarily false: it can represent an overlap (even a highly significant one) of two clones that is not confirmed by other clones (see Fig. AF2**.**10a). An excluded clone may also not necessarily be chimerical: it can just have no “parallel” clone(s) (Fig. AF2**.**10b).


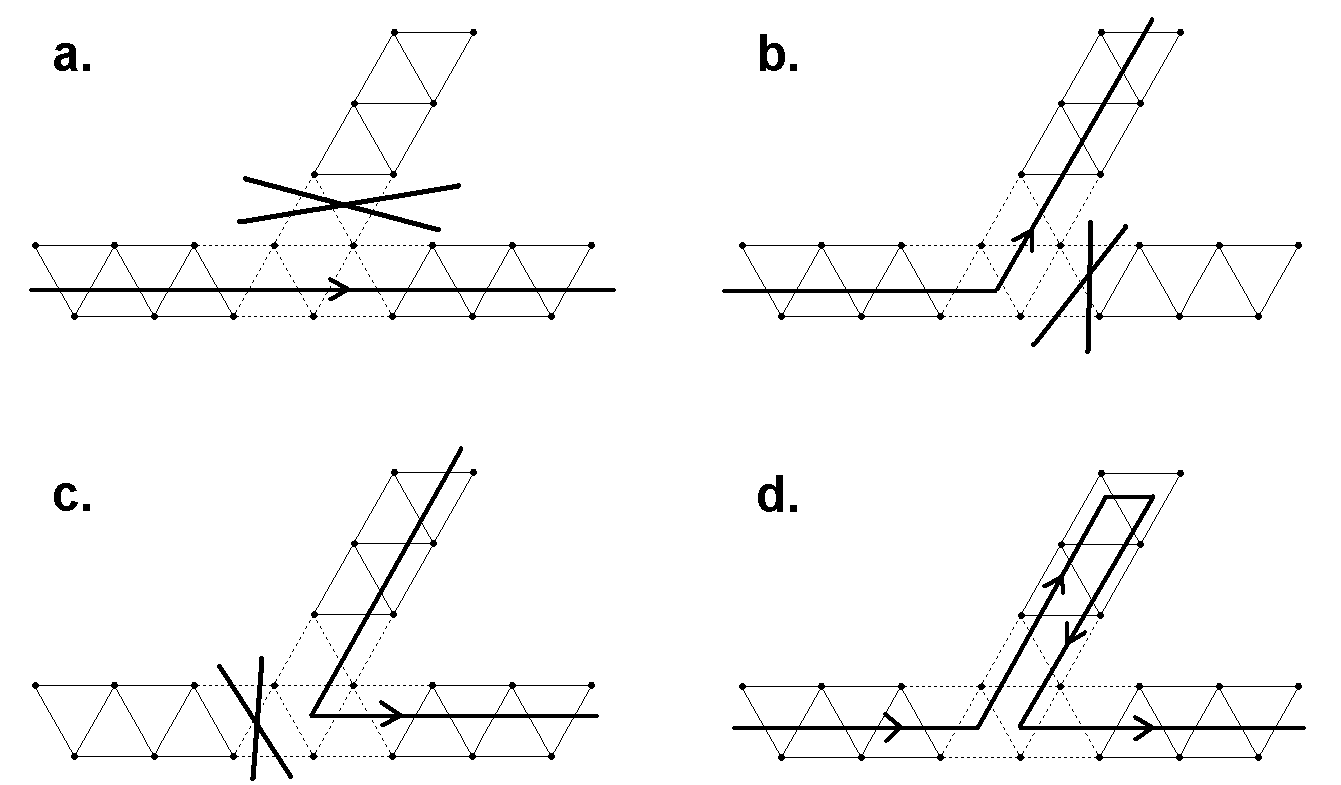


**Fig. AF2.2.** **End-to-end merging of contigs.** Three contigs with linear topological structure (significant clone overlaps marked by solid lines) are end-to-end connected via additional clones and significant clone overlaps (possibly excluded in previous stages, marked by dotted lines). There are several possibilities of merging: (a), (b), (c) end-to-end merging of two contigs (overlaps with clones from the third contigs considered as false significant); (d) merging of all three contigs with reordering clones in the second one.


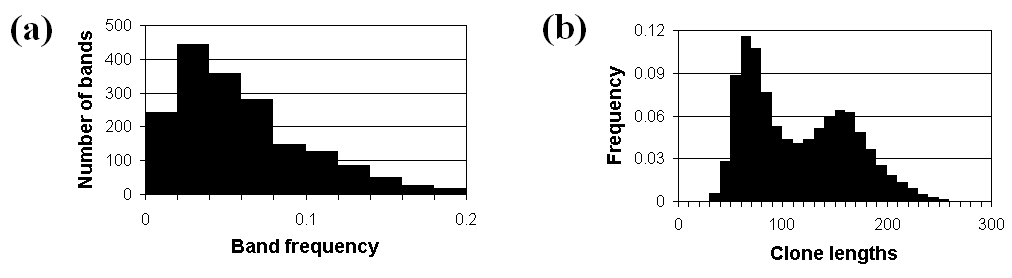


**Fig. AF2.3. Variation in band frequencies and clone lengths in 3B wheat data.** Number of clones *N*=56,952; composed band length is 4,500*4=18,000; constant tolerance=4 was used. **(a)** Band frequencies calculated as fraction of clones having bands with similar (pertaining to tolerance value) length. **(b)** Distribution of clone length. Bimodal form of distribution results from the construction of the BAC library (is not characteristic for other physical mapping projects).

**
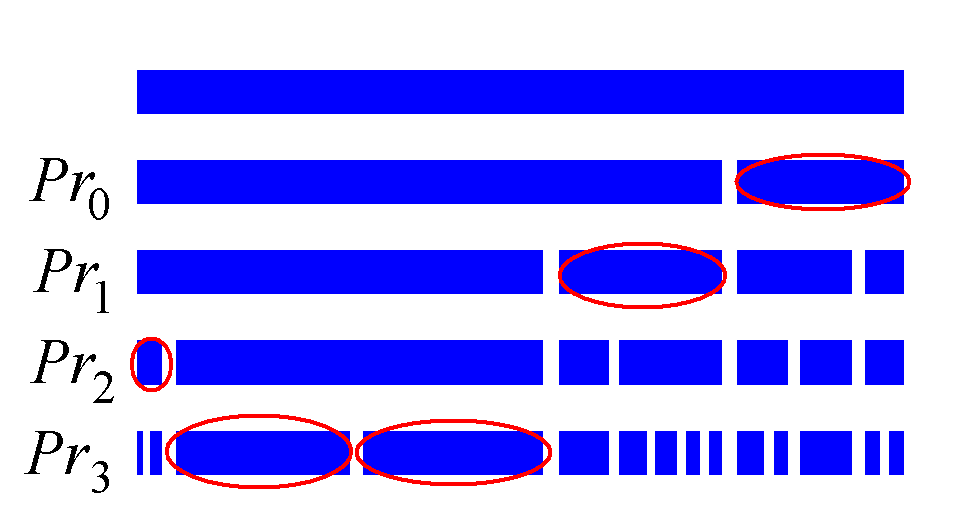
**

**Fig. AF2.4. Clustering with adaptively changing cutoff.** Results of the clustering with four different cutoffs are represented by the division of the rectangle into parts ( (size of sectioned parts represent the number of elements in cluster). Clustering with more stringent cutoffs helps to split large clusters into clusters of reasonable size. Without “protection” (marked by red ellipses) reasonably sized clusters obtained under more liberal cutoff can be “dissolved” under more stringent cutoffs.

**
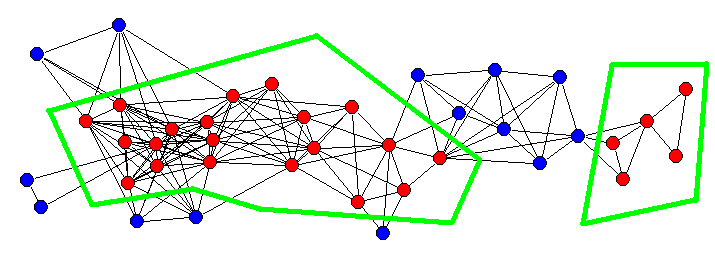
**

**Fig. AF2.5. Repair of gap in FPC contig Ctg888 by adding clones from the database.** Vertices represent clones. Edges represent significant relative to arbitrarily (for certainty) selected cutoff *Pr*_0_(*^SnidM^*)=10^-12^) clone overlaps. Vertices corresponding to clones from FPC contig Ctg888 are marked by **red**. Vertices corresponding to additional clones from BAC library having rank 1 relative to clones from Ctg888 on net of significant clone overlaps are marked by **blue**. Two non-overlapped parts of Ctg888 are marked by **green**. Added clones repair the gap.

**
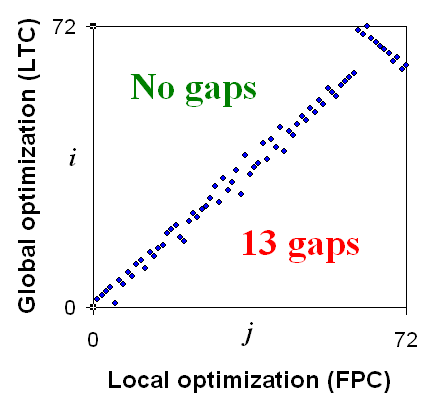
**

**Fig. AF2.6. Order of clones obtained by global optimization methods vs. order of clones obtained by FPC.** Thirteen gaps were detected in FPC order (places where adjacent clones overlapped only non-significantly relative to liberal cutoff *Pr*_0_=10^-12^), while no gaps were found after the reordering of clones using our global optimization procedure.

**
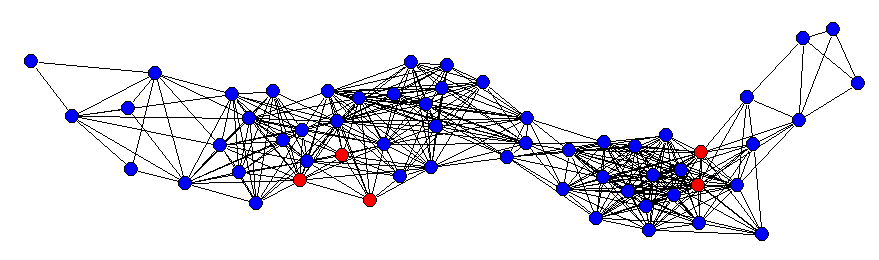
**

**Fig. AF2.7. Example of an FPC contig where the presence of a genetic marker destroys linearity of net structure.** Vertices represent clones. Edges represent significant relative to arbitrary (for certainty) selected cutoff *Pr*_0_(*^SnidM^*)=10^-12^) clone overlaps. Marker BF484536-3B is amplified in clones marked by **red**.

**
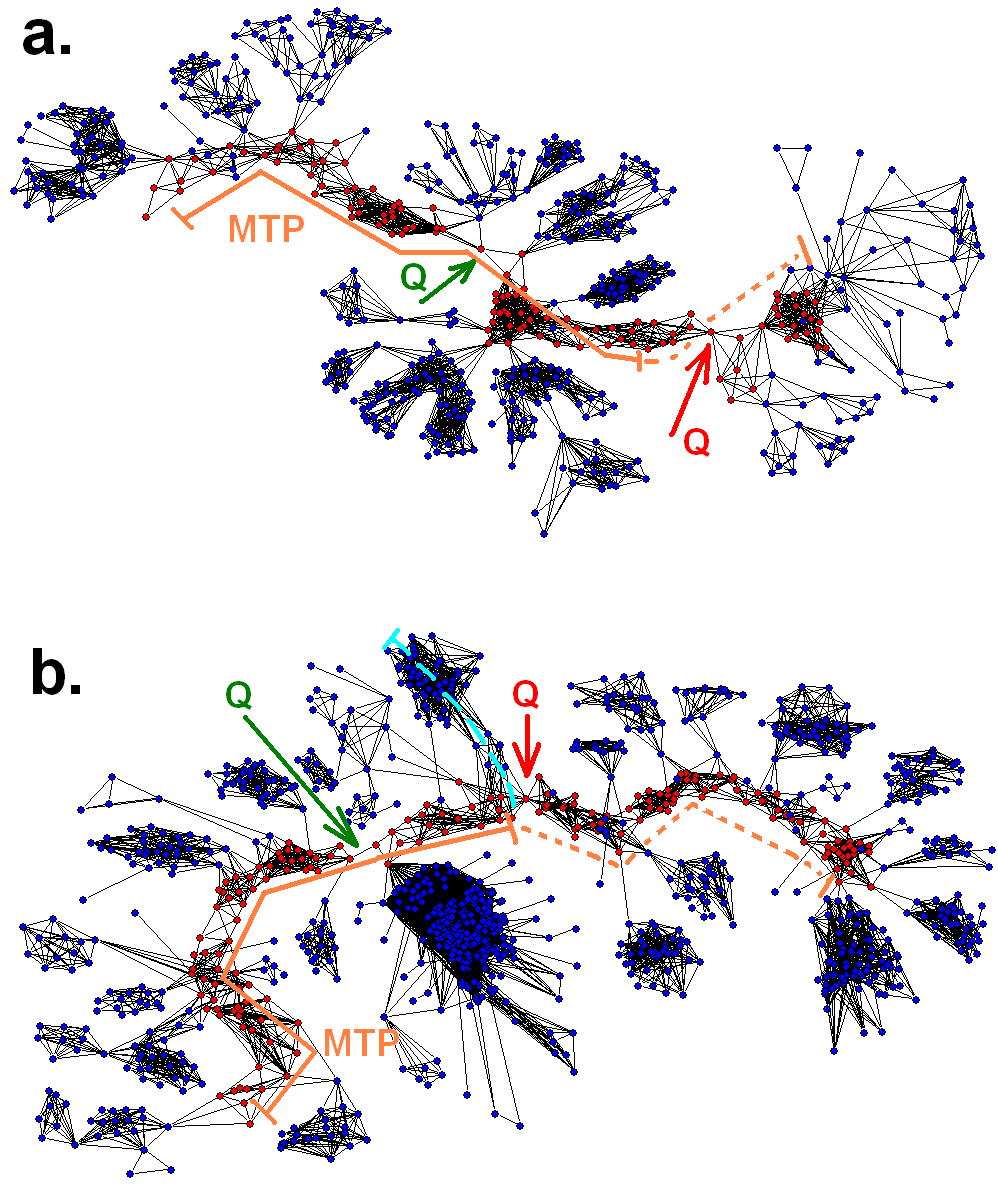
**

**Fig. AF2.8. Examples of FPC contigs where gaps in MTP observed at the sequence level can be explained by problems at the clustering stage.** Vertices represent clones. Edges represent significant relative to arbitrary (for certainty) selected cutoff *Pr*_0_(*^SnidM^*)=10^-12^) clone overlaps. Clones from FPC contig (marked by **red**) are presented together with a set of other clones from 3B BAC library (marked by **blue**). Only clones having ranks 1, 2, and 3 relative clones from the FPC contig are shown. Clones from MTP are not shown but general trace of MTP is presented by the **orange** path. One of the parts proven by sequencing is marked by the solid line. Seemingly, this part cannot be elongated through the whole FPC contig because the continuity of this contig is provided by Q-clones (marked in **red arrow**) are not proven by short parallel paths. **(a)** FPC contig Ctg79 with 117 clones. **(b)** FPC contig Ctg464 with 179 clones. Alternatively, the proven part of MTP for this contig (marked by solid orange line) can be elongated through one of the LTC contigs containing clones from FPC contig Ctg464. The general trace of LTC-based MTP elongation is presented by **light blue** dashed path. Note that although the MTP paths are going here through Q-clone (a) and Q-overlap (b) (marked in **green arrow**) its reality was proven directly via PCR based on BAC-end-sequencing results (see also Fig. AF2**.**10).


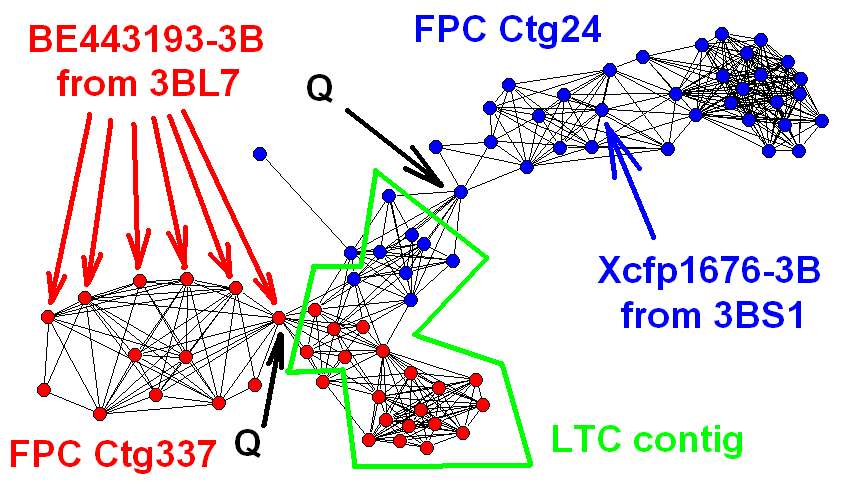


**Fig. AF2.9. Example of a LTC contig containing clones from two FPC contigs, Ctg24 and Ctg337, that were identified as belonging to different chromosomal zones after a genetic markers assignment.** Vertices represent clones. Edges represent significant clone overlaps. Vertices corresponding to clones from FPC contigs Ctg24 and Ctg337 are marked by **blue** and **red,** respectively. Vertices corresponding to clones containing markers are marked by arrows with the name of markers and chromosomal zones. Vertices corresponding to clones from the LTC contig (belonging also to FPC Ctg24 or FPC Ctg337) are contoured by **green**. The difference between FPC and LTC contigs can be explained by the fact that both FPC Ctg24 and Ctg337 contain Q-clones (from the viewpoint of LTC, marked by **black arrows**). Hence, clones from the presented LTC contig do not belong to zones 3BS1 or(and) 3BL7 in contrast to results obtained by FPC.

**
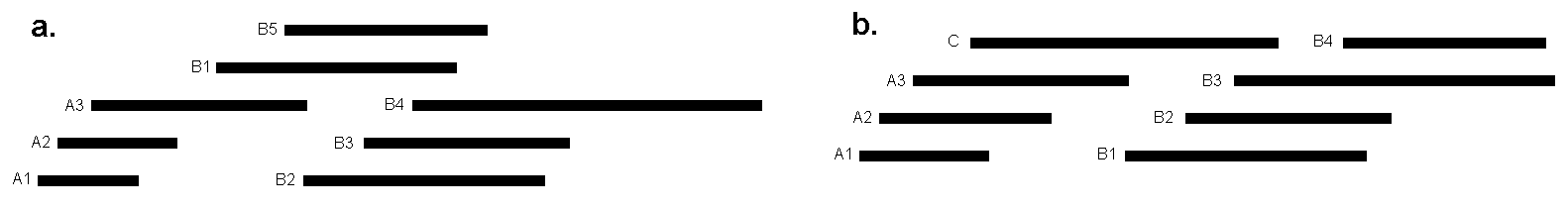
**

**Fig. AF2.10. (a)** Possible interposition of clones within the chromosome corresponding to the net presented in Fig. AF2**.**1a without false significant overlaps: overlap A2B1 is not covered by other clones. **(b)** Possible interposition of clones within the chromosome corresponding to the net presented in Fig. AF2**.**1b without false significant overlaps and chimerical clones: the middle of clone C is not covered by other clones.
